# Bio-Based Aromatic Epoxy Monomers for Thermoset Materials

**DOI:** 10.3390/molecules22010149

**Published:** 2017-01-17

**Authors:** Feifei Ng, Guillaume Couture, Coralie Philippe, Bernard Boutevin, Sylvain Caillol

**Affiliations:** Institut Charles Gerhardt—UMR 5253, CNRS, Université de Montpellier, ENSCM, 8 rue de l’Ecole Normale, 34296 Montpellier, France; feifeing.fr@gmail.com (F.N.); Guillaume.couture@enscm.fr (G.C.); coralie.philippe@enscm.fr (C.P.); bernard.boutevin@enscm.fr (B.B.)

**Keywords:** epoxidation, aromatic, epichlorohydrin, tannin, lignin, cardanol

## Abstract

The synthesis of polymers from renewable resources is a burning issue that is actively investigated. Polyepoxide networks constitute a major class of thermosetting polymers and are extensively used as coatings, electronic materials, adhesives. Owing to their outstanding mechanical and electrical properties, chemical resistance, adhesion, and minimal shrinkage after curing, they are used in structural applications as well. Most of these thermosets are industrially manufactured from bisphenol A (BPA), a substance that was initially synthesized as a chemical estrogen. The awareness on BPA toxicity combined with the limited availability and volatile cost of fossil resources and the non-recyclability of thermosets implies necessary changes in the field of epoxy networks. Thus, substitution of BPA has witnessed an increasing number of studies both from the academic and industrial sides. This review proposes to give an overview of the reported aromatic multifunctional epoxide building blocks synthesized from biomass or from molecules that could be obtained from transformed biomass. After a reminder of the main glycidylation routes and mechanisms and the recent knowledge on BPA toxicity and legal issues, this review will provide a brief description of the main natural sources of aromatic molecules. The different epoxy prepolymers will then be organized from simple, mono-aromatic di-epoxy, to mono-aromatic poly-epoxy, to di-aromatic di-epoxy compounds, and finally to derivatives possessing numerous aromatic rings and epoxy groups.

## 1. Introduction

Amidst materials widely used in plastic industry nowadays, thermosets (or thermosetting polymers) represent about 20% of plastic production [1]. They are formed from a liquid or solid mixture of various ingredients including at least one or more monomers. One of these monomers at least exhibits a functionality equal or higher than three, thus enabling the creation of a solid three-dimensional non-fusible network via an external action such as heating or UV irradiation [2]. Thermosets include a wide range of reactive systems such as phenolic and urea formaldehyde resins, unsaturated polyesters, and polyepoxides, the latter accounting for nearly 70% of the market. Polyepoxides are one of the most versatile class of compounds with diverse applications, especially coatings, which dominate the market, but also water containers, automotive primer, printed circuit boards, semiconductor capsules, adhesives, and aerospace composites. The global production of epoxy prepolymers is estimated to reach 3 million tons by 2017 for a market of US$ 20 billion in 2015. This success arises from the excellent mechanical strength and toughness, outstanding chemical, moisture, and corrosion resistance of the epoxy thermosets [3,4]. This list does not include various interesting process-related characteristics such as: the absence of volatile products emitted during the polymerization reaction, the large choice of monomers available or the high adhesion properties to a variety of surfaces.

Over 90% of these epoxy materials are based on bis(4-hydroxyphenylene)-2,2-propane, known as bisphenol A (BPA), a petrol-based molecule first synthesized in the 1890s and used as a synthetic oestrogen [5]. Aromatic compounds are widely used in organic materials for their stability, their toughness, and above all their ability to structure the matter by π-stacking, thus allowing BPA to confer good thermal and mechanical properties to the epoxy thermosets. Commercialized for more than 50 years, BPA, mainly through its epoxy form DiGlycidylEther of Bisphenol A (DGEBA), is nowadays spread in many coatings, adhesives, laminates and composites, but also many domestic or even health related-products such as plastic bags, food containers and metal cans, dental sealants, soaps and lotions [6,7].

However, epoxy thermosets are sensitive to hydrolysis, which may cause BPA to leach, leading to widespread human exposure [6,7,8,9]. Unfortunately, it has been classified as carcinogen mutagen and reprotoxic (CMR), and is recognized as an endocrine disruptor [10]. Consequently, many governments have recently hardened the legislation regarding the production and use of BPA, especially in baby’s bottles, food containers and medical supplies [11,12]. Moreover, BPA is synthesized from oil-based phenol and the 3D chemically crosslinked networks of thermosets prevent them from being recycled by heating. The awareness on BPA toxicity combined with the limited availability and volatile cost of fossil resources, and the non-recyclability of thermosets implies necessary changes in the field of epoxy networks. Thus, substitution of BPA has witnessed an increasing number of studies both from the academic and industrial sides. Over the past decades, many bio-based resources have been tested as potential candidates for replacing BPA in epoxy resins, but very few of them have reached the commercialization step. Among these, epoxidized natural oils [13,14,15] and modified cardanol [16] are the only two types of epoxy resins based on natural and non-toxic precursors, commercially available in the market. However, the low reactivity of the epoxy groups along their aliphatic backbone [1] and the low glass transition temperature caused by the alkyl chain prevent them from competing with BPA-based materials with their high *T*_g_ values and glassy moduli [17]. For this reason, many researches have been devoted to using glycidylated aromatic bio-based materials as substitutes for BPA. Very interesting and exhaustive reviews have recently been published on bio-based precursors for thermosets and their hardeners [1,18,19,20,21], but to our knowledge, none of them focuses especially on aromatic epoxy monomers based on biomass resources. In fact, only aromatic poly-epoxides seem to be able to compete with DGEBA in terms of thermo-mechanical properties, making them of primary interest for renewability.

Thus, the present review proposes to give an overview of the reported aromatic multifunctional epoxide building blocks synthesized from biomass or from molecules that could be obtained from transformed biomass. After a reminder of the main glycidylation routes and mechanisms and the recent knowledge on BPA toxicity and legal issues, this review will provide a brief description of the main natural sources of aromatic molecules. The different epoxy prepolymers will then be organized from simple, mono-aromatic di-epoxy, to mono-aromatic poly-epoxy, to di-aromatic di-epoxy compounds, and finally to derivatives possessing numerous aromatic rings and epoxy groups. For each one, the curing agent used and the thermal properties (especially *T*_g_ and thermal degradation) of the crosslinked material will be given along with their DGEBA-based counterparts if available. The potential toxicity of the epoxy precursors will also be mentioned. Tables gathering all these results will be given at the end of each part for comparative purposes.

## 2. Epoxidation Methods and Processes

**Scheme 1:**
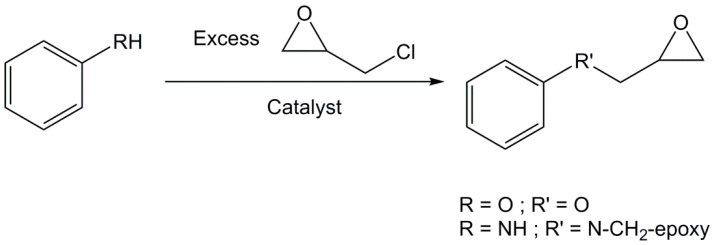
Synthesis of epoxy derivatives from phenol or aniline by direct glycidylation with epichlorohydrin.

**Scheme 2:**
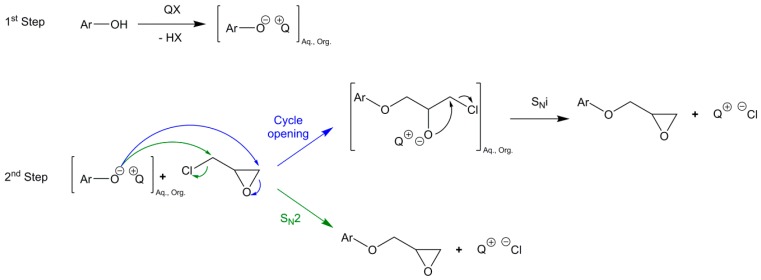
Mechanism of coupling between phenolic compounds and epichlorohydrin (ECH) in the presence of a phase transfer catalyst (QX) [1,27].

Poly-functional epoxy compounds are very reactive building blocks and can lead to materials by chain-growth polymerization or crosslinking with anhydrides, phenols and amines. They are generally prepared by direct glycidylation, as showed in Scheme 1 [1,22,23]. It consists in reacting an alcohol or amine derivative with epichlorohydrin (ECH) in the presence of an alkylammonium halide as a phase transfer catalyst, such as benzyltriethylammonium chloride, tetrabutylammonium bromide or cetyltrimethyl ammonium chloride. Sometimes, epibromohydrin is used instead of its chloride analogue [24]. A sodium or potassium hydroxide post-treatment is usually applied in same pot to increase the number of epoxy rings. In fact, the phenolic oxygen may displace the chlorine atom to directly yield the desired product via a S_N_2 mechanism, or open the epoxy ring causing the formation of a chlorinated derivative that can be closed by a strong base through a S_N_i mechanism (Scheme 2) [25,26]. S_N_i stands for Substitution Nucleophilic internal and is a nucleophilic substitution mechanism that implies a retention of configuration.

**Figure 1:**
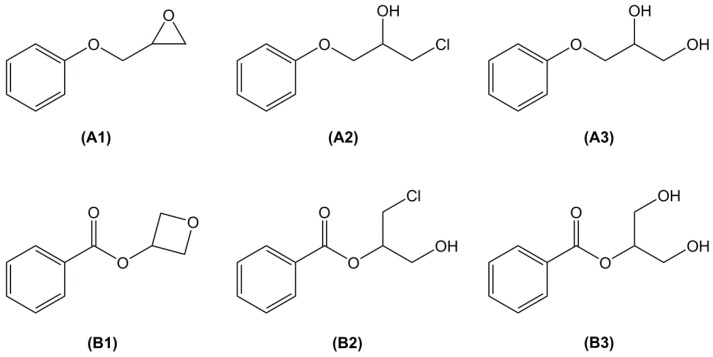
Common side-products of the direct glycidylation of phenol (**A1**–**A3**) and benzoic acid (**B1**–**B3**).

**Scheme 3:**
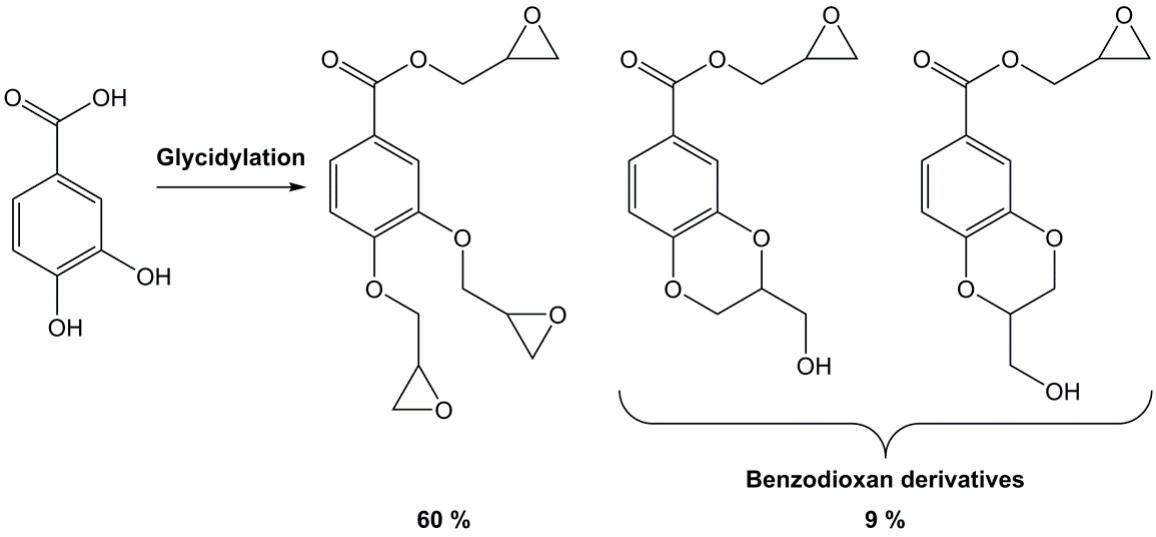
Products obtained during the glycidylation of protocatechuic acid with epichlorohydrin [38].

The direct glycidylation of phenol has proven to yield several possible side products [28,29,30,31,32] including chlorinated and diol derivatives (Figure 1). Furthermore, the higher reactivity of benzoic acid may lead to an opening of the epoxy via both carbon atoms, leading to new chlorinated (**B2**), diol (**B3**) and oxetane ring (**B1**) side-products [33,34,35,36,37]. Another important side-product observed during the glycidylation step with epichlorohydrin is a benzodioxan derivative obtained by an intra cyclization occurring with two phenolic groups in *ortho* position (Scheme 3).

**Scheme 4:**
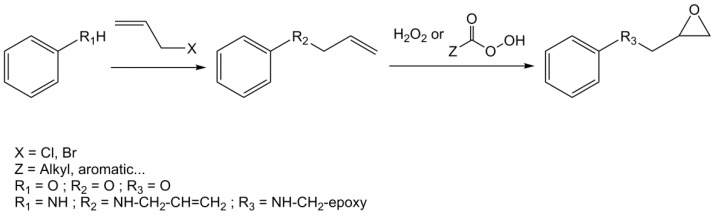
Synthetic pathway to obtain epoxy from phenol or aniline using allyl halide and oxidation.

To avoid these drawbacks, Meurs et al. [39] developed a process to obtain glycidyl derivatives from phenol, glycidol and propylene carbonate but it requires the use of high temperatures in autoclave and relatively harsh conditions. A two-step synthesis can also be used to form epoxy compounds: it involves the *O*- or *N*-allylation of the corresponding alcohol or amine derivatives using an allyl halide, followed by the oxidation of the resulting double bond (Scheme 4). Unfortunately, allyl chloride and allyl bromide are both toxic derivatives. Moreover, hydrogen peroxide exhibitis low reactivity toward allyl ether oxidation except at high concentrations or in the presence of metal transition catalysts [40]. The use of stronger, more toxic peracids such as *m*-chloroperbenzoic acid (*m*CPBA) is sometimes considered, but Aouf et al. [22] found that an excess of peracid is also required and the *m*-chlorobenzoic acid formed during the oxidation of allylated gallic acid is difficult to eliminate. The epoxidation of allyl groups by potassium peroxymonosulfate (also known as Oxone) can be considered a sustainable pathway. It is based on the Shi epoxidation, which uses a fructose-derived organocatalyst with Oxone and ketones to generate in situ dioxiranes [41], which are strong epoxidation agents. However, the epoxidation of electron-deficient alkenes such as allyl groups by dioxiranes can be very slow [42,43,44]. The reaction may require the use of ketones bearing highly electroattractive groups such as 1,1,1-trifluoroacetone to increase the overall yield [22,45]. Enzymatic catalysts have also been developed as greener alternatives for the oxidation of carbon-carbon double-bonds. First developed to replace the Prileshajev reaction applied at an industrial scale to produce epoxy vegetable oils [46], one of these catalyst (immobilized lipase B from *Candida antarctica* (Novozym 435)) has also been used by Aouf et al. [40] to obtain epoxy gallic acid and vanillic acid from their allylated precursors in high yields.

**Scheme 5:**
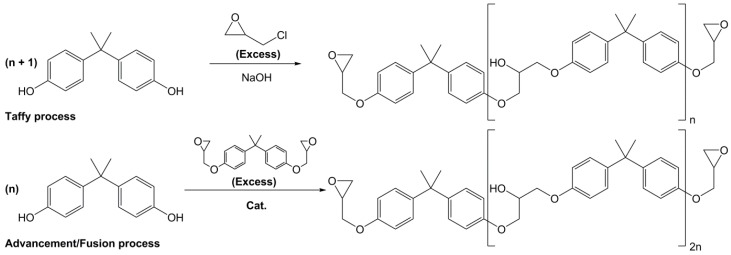
Two main industrial processes for the synthesis of monomers and oligomers of diglycidyl ether of vanillyl alcohol (DGEBA): (i) the taffy process with a controlled excess of epichlorohydrin and (ii) the advancement/fusion process using an excess of a pre-synthesized DGEBA monomer.

Overall, the direct glycidylation remains the main synthetic pathway used for the industrial synthesis of Diglycidyl Ether of Bisphenol A, as it allows the recovery of both monomers and oligomers for tunable properties [23,47]. By reacting bisphenol A with a controlled excess of epichlorohydrin, the “taffy” process yields either monomers or short oligomers of DGEBA (Scheme 5). To increase the chain length of the oligomers, “advancement” (with solvent) or “fusion” (without solvent) processes can be chosen. They both consist in reacting BPA with an excess of a pre-synthesized DGEBA monomer to extend the chain. “Fusion” process is generally preferred for the industrial production of DGEBA oligomers as the purification steps are easier and the chlorine content of the final product is lower than in the case of the “taffy” process, which requires an excess of epichlorohydrin. 

In terms of sustainability, both bisphenol A and epichlorohydrin used for the synthesis of DGEBA are mostly oil-based. BPA is obtained from reaction of acetone and phenol and epichlorohydrin is synthesized in two steps by reacting hypochlorous acid on allyl chloride and then treating the alcohol mixture obtained with a strong base [48]. In 2007, Solvay designed the EPICEROL^TM^ process to produce epichlorohydrin from bio-based glycerol, thus allowing to reduce the content of fossil resources used for DGEBA production [49,50,51,52,53]. However, the percentage of carbon atoms in the oligomers coming from ECH is low, thus making the impact limited. Furthermore, whatever synthetic pathway or process is chosen, reagents (e.g., epichlorohydrin and allyl halides) are all carcinogen agents (H350). Allyl bromide is also very toxic for the environment. The toxicity of these reactions is a true issue, and alternatives have to be found over time. Although the production of epoxides is generally well-controlled by the manufacturer and the resulting resins and materials do not exhibit the toxicity of these reactants, the intrinsic toxicity of BPA remains.

## 3. Toxicity of Bisphenol A and Regulations

Bisphenols (BPs) are part of the common class of endocrine disruptors because of their significant hormonal activity, with Bisphenol A being the most famous of them. In fact, BPA exhibits one of the highest production volume of chemicals worldwide [7], with a manufacture of approximately 3.8 million tons per year in 2006 [54]. About 80% of the global production of BPA is used for the synthesis of polycarbonate, 18% for epoxy resins and the remainder for other applications, such as food containers, paper products (e.g., thermal receipts), water pipes, toys, medical equipment, and electronics [6,7]. As a consequence, human beings and environment are constantly exposed to BPA: it has been detected in 95% of human urine samples, which indicates that this compound may leach into food or water [7,8,9,55]. Furthermore, several studies found that BPA is present in high prevalence in fetuses and infants [7,8,56] and has undeniably an impact on human health leading to precocious puberty, cancer, diabetes, obesity, neurological disorders, and so on. Theoretical and experimental studies can be carried out to forecast the potential toxicity of a compound or to determine the different types of interactions between this compound and estrogen receptors. For example, the “read-across” method, based on analogies between substances is currently developing. It first consists in gathering data on the physical and biological properties of chemicals exhibiting a structure similar to the target molecule. Then, by taking into account previously observed trends, it is considered possible to extrapolate on the target molecule’s behavior [57]. QSAR (Quantitative Structure Activity Relationship) models enable a qualitative and quantitative determination of the endocrine activity in terms of affinity, and give some information about the underlying mechanism [58]. Recently, Delfosse et al. [59] described for the first time the mode of action of BPA at the molecular scale and developed a bio-informatics tool to predict the interactions between bisphenols and the target receptors (estrogen receptors (ERs) or other members of the nuclear hormone receptor family). Numerous studies demonstrated that bisphenol A has two major modes of actions: steroid related mode and epigenetic mode. Concerning the latter, Dolinoy et al. [60] have shown the ability of BPA to alter DNA methylation. Regarding the steroid related mode, BPA can act as an estrogen agonist when it binds to nuclear estrogen receptors (ERα and ERβ) [61,62,63,64]. More recently, Takayanagi et al. [65] demonstrated that BPA also binds to the estrogen-related receptor-γ (ERRγ) with high constitutive activity. Furthermore, BPA also behaves as an androgen receptor antagonist (AR) which affects the activation and function of the AR [66,67]. According to many lines of evidence, BPA acts as an endocrine disruptor even at low doses. A review, based on hundreds of studies, concluded that there is sufficient evidence for low dose effects of BPA. Indeed, for these studies, authors used doses below those used for traditional toxicological studies, and found nonmonotonic dose-response curves [68].

**Figure 2:**
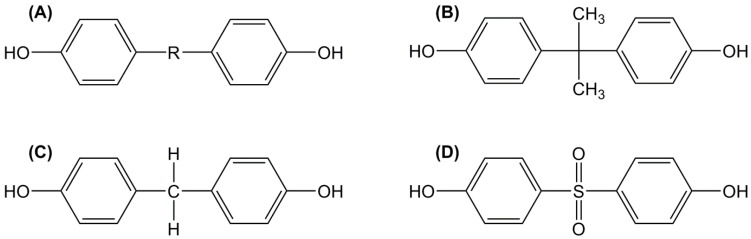
Chemical structures of bisphenol analogues (**A**); bisphenol A (**B**); bisphenol F (**C**) and bisphenol S (**D**).

**Figure 3:**
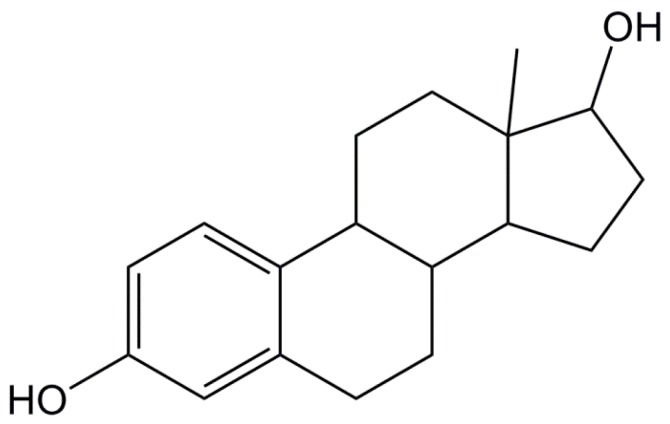
Structure of 17β-estradiol.

Bisphenols are composed of two phenols linked by a central carbon atom, which in the case of BPA bears two additional methyl groups (Figure 2B). These structural features make BPA able to mimic the natural estrogen 17β-estradiol (Figure 3), in terms of binding ability to estrogen receptors [69,70]. In fact, the main characteristics of the natural ligands required for the steroid activity are the presence of phenol groups on a hydrophobic backbone [71,72]. A recent paper has demonstrated that all the structural elements of BPA are prerequisite for binding the estrogen-related receptor-γ (ERRγ), especially the two phenolic and methyl groups [10]. Firstly, the authors demonstrated that the phenol structure of BPA is an essential element to bind ERRγ and only one of the two phenolic hydroxy groups is required for the full binding. Nevertheless, the presence of a second oxygen-based group increases the steroid activity [73].

**Figure 4:**
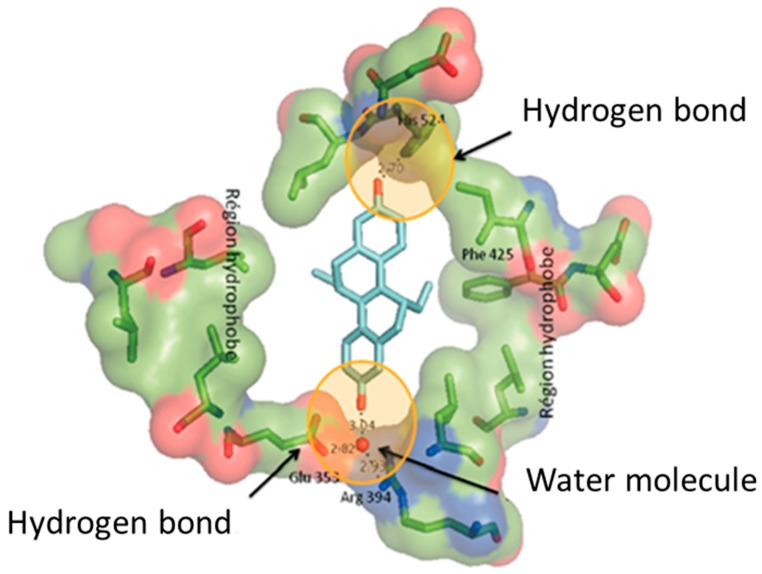
X-ray structure of the hydrophobic pocket of estrogen receptor (ER)α [76], according to the works of Brzozowski et al. [74] and Tanenbaum et al. [75].

By analogy with 17β-estradiol, these two hydroxyl groups are essential to establish interactions with the hydrophobic pocket created by the receptor [74,75]. This pocket consists of several combining sites where estrogen or other ligands can bind. The size of the pocket is 440 Å [74,75,76], which is bigger than the natural estrogen molecule size (245 Å) allowing hydrogen binding interactions (Figure 4). Liu et al. [77] also showed that the substitution of one of the two aromatic rings by a methyl or ethyl group led to a decrease of the interaction with the hydrophobic part of the receptor, whereas the presence of chlorine substituents on the 3–5 positions of the first aromatic ring strengthens affinity with the receptor.

The presence and the distance between the two hydroxyl groups are not the only critical factors. Okada et al. [10] clearly demonstrated that the alkyl groups on the central carbon atom of bisphenol play a key role in selection of the human estrogen receptors: ERRγ or ERα. ERα prefers the bulkier and more electrophilic alkyl groups, whereas ERRγ prefers the less bulky and less electrophilic alkyl groups. A bulky group on the central carbon atom is obviously disadvantageous in terms of binding BPA to ERRγ’s binding pocket and reduces its activity. Furthermore, it was shown that one of the two methyl groups on the central carbon atom of BPA is involved in the hydrophobic intermolecular interaction with the receptor residue, such as CH_3_-alkyl and CH/π interactions.

The increasing concerns about the detrimental effects of BPA has led governments to enforce regulations mostly in the European Union and North America in order to limit the exposition of their citizens to this substance. For example, in 2014, in line with the opinion adopted by the RAC (Risk Assessment Committee), Bisphenol A has been classified in the hazard class reproductive toxicity category 1B “may damage fertility”. In the frame of the REACH regulation, the French proposition of the restriction of BPA in thermal papers was approved on the 6 July 2016 by the REACH Committee. The BPA European legislation involves different elements such as the restriction of the contact of infants and young people with this substance, through toys, feeding bottles, etc. [11,78,79,80]. BPA is authorized as additive or monomer in the manufacture of plastic materials and articles in contact with food and water but a specific migration limit value of 0.6 mg/kg of food has been set [12]. Regarding cosmetic products, Bisphenol A is recorded in the list of the prohibited substances [81]. All the products made of BPA can’t be eligible for a positive Eco-Label [82] and the indicative limit of occupational exposure [83] to BPA particles is 10 mg/m^3^. Some countries such as France have established even more strict legislations towards BPA [84], and this country proposed BPA as a REACH Regulation candidate substance of very high concern (SVHC) [85] and confirmed its advert on the 30 August 2016.

Following the public concern and the stringent regulations on the production and use of BPA, several bisphenol analogues have been produced as alternative substances. These following analogues, BPF (4,4′-methylenediphenol) and BPS (4-hydroxyphenyl sulfone) (Figure 2C,D), are frequently found in most scientific and environmental studies because they are among the main substitutes of BPA in polycarbonate-based plastics and epoxy resins. Available studies have reported a variety of detrimental effects of these bisphenol analogues [86] and showed that the toxicity of these analogs is similar to or even greater than that of BPA [87,88]. The toxic effects include endocrine disruption, cytotoxicity, genotoxicity, reproductive toxicity, neurotoxicity, etc. A recent article by Rochester and Bolden [89], focusing on the hormonal activities of BPF and BPS demonstrated that these two analogues have a hormonal action similar to BPA in vitro and in vivo. Hexafluorobisphenol A (BPAF) and 4,4′-(1-methylpropylidene) bisphenol (BPB), two others substituents of BPA, have also shown estrogenic and anti-androgenic activities [88]. Therefore, a harmless let alone renewable epoxy building block is still required [90]. For this reason, bio-mass has been considered a cheap source of potentially functionalizable monomers or oligomers. As previously stated, the already commercialized bio-based epoxy monomers cannot compete with DGEBA-based materials in terms of thermo-mechanical properties, because of their characteristic structure and low aromatic content. Thus, the next part will briefly present the natural sources of renewable aromatic compounds potentially capable of standing up for BPA replacement.

## 4. Main Natural Sources of Aromatic Moieties

The present part will briefly summarize the main sources of aromatic moieties bearing reactive groups suitable for the introduction of epoxy moieties. This summary will include resources naturally containing small, phenolic compound such as eugenol extractible from plant natural oils, or polyphenolic crosslinked polymers such as tannins and lignin that can be directly functionalized or depolymerized into smaller molecules prior to epoxidation steps. Some information on their availability or worldwide production will be given as well as the main known characteristics of their structure. The reader may refer to the vastly documented review from Lochab et al. [91] and book from Belgacem and Gandini [92] on renewable resources and naturally occurring phenolic derivatives to obtain more detailed data.

### 4.1. Lignin

**Figure 5:**
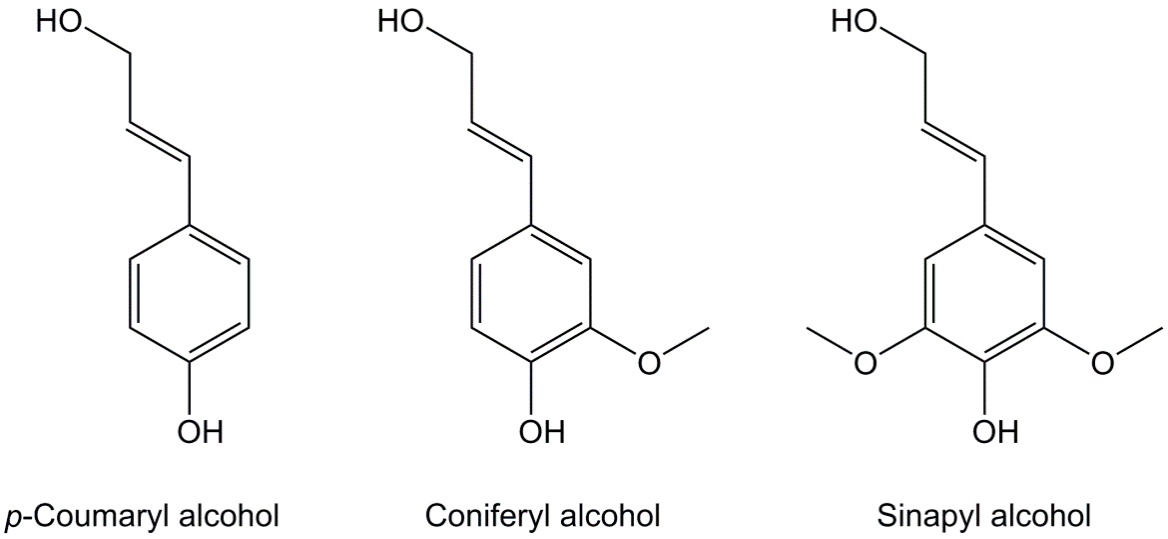
Main aromatic subunits found in lignin.

Lignin is the widest distributed aromatic biopolymer and the second most abundant naturally occurring macromolecule after cellulose, constituting from 1% to 43% by weight of the dry lignocellulosic biomass, with a potential availability exceeding 300 billion tons [17,93,94,95]. It is a cell-wall component bonding cells together in the woody stems, providing them with their well-known rigidity and impact resistance. Although its absolute structure remains unknown and varies according to the plant it originates from and its environment, lignin is an amorphous three-dimensional polymer network of three main methoxylated phenyl propane units (Figure 5) with seven major linkages: β-*O*-4 (aryl ether), α-*O*-4, β-β (pinoresinol), β-5 (phenylcoumaran), β-1 (diphenylmethane), 5,5 and 4-*O*-5 (diphenyl ether) linkages. It exhibits various functional groups such as aliphatic and phenolic hydroxyl, carboxylic, carbonyl and methoxy moieties.

**Figure 6:**
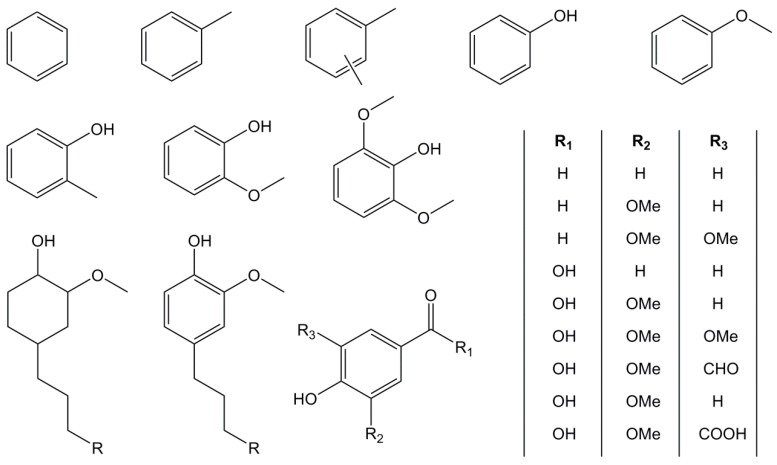
Various aromatic moieties obtained from lignin depolymerization [93,94].

Usually, lignin is viewed as a waste material derived from the wood pulp in the paper industry and available in large quantity (50–70 million of tons estimated) [96]. Unfortunately, only 1% to 2% of overall lignin is used for more specific applications, the remaining primarily serving as a (bio)fuel for the cellulose extraction [97]. Lignin is extracted from lignocellulosic biomass by two main categories of processes: (i) sulfur processes yielding lignosulfate and Kraft lignin and (ii) sulfur-free processes yielding organosolv and soda lignin. These extraction methods greatly influence the already complex structure of the polymer, sometimes making it difficult to directly use it as a chemical precursor. For this reason, works have been carried out to depolymerize lignin into smaller, simpler aromatic molecules suitable for chemical modification and/or polymerization. For example, when lignin is depolymerized, compounds such as vanillin, phenols derivatives, cresols, ferulic and coumaric acids are released (Figure 6). All these molecules are already oil-based but this pathway offers an interesting solution for their renewability, thus increasing thermosets renewability.

### 4.2. Tannins

Tannins are the second bio-based source of natural phenolic moieties and the third most abundant compounds extracted from wood biomass, with 160,000 tons bio-synthesized each year [98,99]. They can be found mainly in the soft tissues such as wood, bark, leaves or needles of all vascular and some non-vascular plants, in which they play a protective role against outside aggressions and in plant growth regulation [100]. Tannins are polyphenol derivatives with low molecular weights and can be divided into three main categories: hydrolysable tannins, condensed tannins and complex tannins, the latter being a combination of the first two.

**Figure 7:**
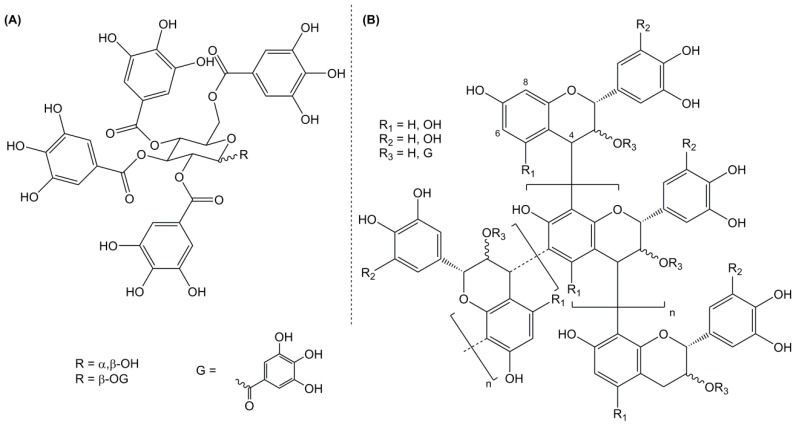
Main structures of tannins from vascular plants: hydrolysable tannins (**A**) and condensed tannins (**B**).

Hydrolysable tannins are a mixture of phenolic esters of sugars (Figure 7A) readily hydrolyzed by acids, alkalis or enzymes, and present a low availability (less than 10% of the world’s commercial production) [101]. They are mainly used in the tanning industry. The condensed tannins may represent the most interesting derivatives with 90% of global production. They are based on four types of repeating flavonoid units: profisetinidin, procyanidin, prorobinetidin, and prodelphinidin linked by C4–C6 or C4–C8 bonds (Figure 7B). Thanks to their availability, aromatic moieties and rigid structure, their numerous functionalizable hydroxyl functions and nucleophilic sites, condensed tannins may represent an interesting candidate for BPA substitution. Similarly to lignin, some studies are conducted on the depolymerization of tannins to obtain phenolic monomers as building units for thermosets [98,102,103]. For example, Roumeas et al. [102] successively used thiol and furan derivatives as nucleophiles for the acid-assisted depolymerization of condensed tannins into catechin and thioether or furan derivatives of catechin.

**Figure 8:**
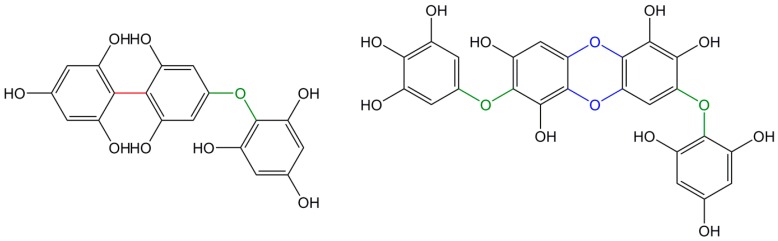
Example of phlorotannins linkages with phenyl bonds (in red), ether bonds (in green) and dibenzo-*p*-dioxin bond (in blue).

Finally, a last class of tannins, phlorotannins, can be found in non-vascular plants such as algae and are based on polymerized phloroglucinol (1,3,5-trihydroxybenzene) with a large range of molecular weights [98]. They play a role similar to condensed tannins in vascular plant and can as well be divided into categories according to the link between phloroglucinol units e.g., ether, phenyl bonds, a combination of both, or a dibenzo-*p*-dioxin bond (Figure 8).

### 4.3. Cardanol

**Figure 9:**
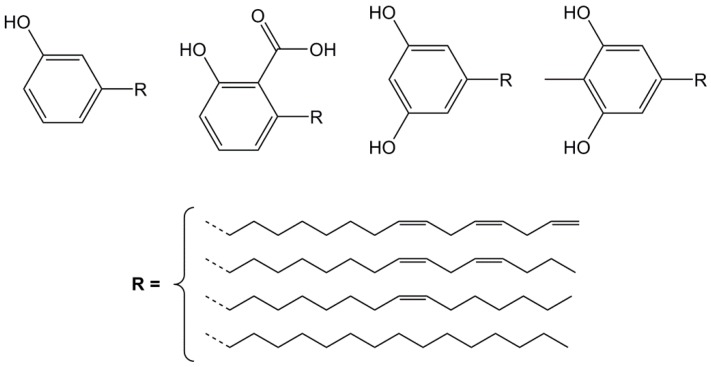
Main phenolic constituents of cashew nut shell liquid (from left to right): cardanol, anacardic acid, cardol and 2-methyl cardol [104].

Cashew nut shell liquid (CNSL) is a reddish-brown liquid that can be extracted from the soft honeycomb structure located inside the cashew nut shell and constitutes from 30 to 35 wt % of it [16,91,104]. With approximately 2.1 millions of tons of cashew nuts produced every year, mainly from countries in Asia (India, Vietnam) and Africa (Nigeria, Ivory Coast), CNSL represents an abundant non-edible by-product that has already found various applications in coatings, laminates and adhesives to name a few. However, it also represents an interesting source of aromatic chemical building blocks. In fact, CNSL is mainly composed of four major phenolic derivatives: anacardic acid, cardanol, cardol and 2-methyl cardol (Figure 9), the percentage of which depends on the extraction method. Among these, cardanol is often regarded as the most interesting compound, as its percentage can reach 60% in some CNSL grades. Its structure is defined as a mono-aromatic phenol substituted in meta-position by a C15 alkyl chain, on which none to three unsaturations in C8, C11 and C14 are possible [105,106,107]. Thanks to its hydroxyl function and carbon-carbon double bond(s), cardanol can offer various functionalization opportunities, although its long aliphatic chain may severely impact materials in terms of thermo-mechanical properties.

### 4.4. Cellulose and Hemi-Cellulose

**Scheme 6:**
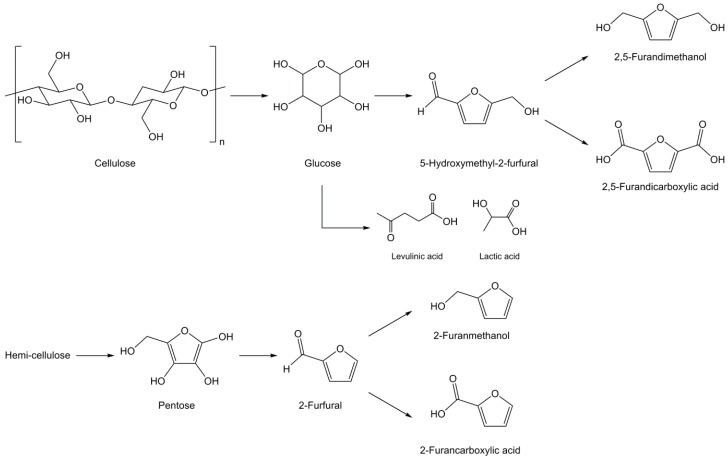
Synthesis of furan derivatives from cellulose and hemi-cellulose [112,113,114].

As previously stated, cellulose is the most abundant naturally occurring polymer and accounts for approximately 40–45 wt % of lignocellulosic biomass depending of wood species [108,109,110,111]. With an annual production estimated around 100 billion tons, it is a remarkable feedstock for the synthesis of renewable chemical building blocks. Cellulose is a crystalline high molecular weight polysaccharide (7000 to 15,000 monomeric units) formed of d-glucose linked by β-1,4-glycosidic bonds. The high chain organization through hydrogen bonding provides cellulose with interesting mechanical properties as well as poor solubility in water and various organic solvents. However, the several hydroxyl groups along its backbone make cellulose highly hydrophilic. Although it doesn’t exhibit an aromatic structure, aromatic building blocks can be obtained from cellulose via depolymerization (Scheme 6) [112,113,114]. Through acid hydrolysis under harsh conditions or bacterial degradation [115] cellulose yields glucose that can further isomerize into fructose and then be dehydrated into 5-hydroxymethyl-2-furfural (HMF), a furanic aldehyde. HMF can also be oxidized into 2,5-furandicarboxylic acid or reduced into 2,5-furandimethanol, two symmetric di-functional aromatic moieties.

The other main component of lignocellulosic biomass, namely hemi-cellulose, is an amorphous polysaccharide with short chains of 500 to 3000 monomer units with acidic groups. Similarly to cellulose, it can be depolymerized into pentose that can further be transformed into 2-furfural, 2-furanmethanol and 2-furancarboxylic acid. However, these derivatives are mono-functional, thus making them unsuitable for the synthesis of di-epoxy monomers, only fit for reactive diluents. Finally, it is also worth noting that cellulose can also be degraded into other interesting, although non aromatic, building blocks: levulinic and lactic acid. 

### 4.5. Other Natural Sources of Aromatic Moieties

**Scheme 7:**
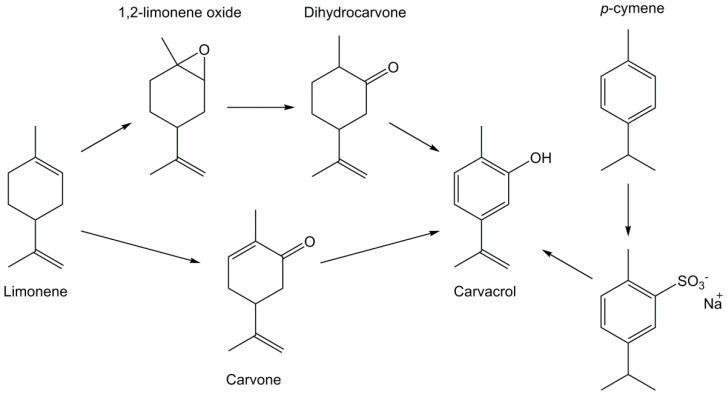
Synthesis of carvacrol from limonene or *p*-cymene according to Harvey et al. [117].

Some other abundant biomasses are potential candidate to extract or synthesize phenolic derivatives. For example, terpenes and terpenoids can be obtained from various plants’ essential oils or as by-products of industrial processes and are largely available at reasonable prices [116,117,118]. For example, 700 millions of kg of limonene are produced annually as a side-product of orange juice production [117]. Terpenes form a large and diverse class of molecules based on repeating isoprene units that can be linked head to tail or form cycloaliphatic or aromatic rings such as in α-pinene found in pines, limonene from citrus fruits or *p*-cymene extracted from thyme, only to name a few. Some of them may represent interesting candidates for the synthesis of epoxy monomers such as carvacrol, which exhibits a phenol moiety and a carbon-carbon double bond (Scheme 7) and can be isolated from oregano and thyme essential oils. However, not all terpenoids contain aromatic and/or phenolic moieties, but these requirements can be reached via different synthesis steps. For example, carvacrol can be obtained from other turpentine components such as limonene via an oxidation followed by an isomerization with sulfated zirconia or from *p*-cymene by the action of concentrated sulfuric acid and sodium hydroxide [117].

**Figure 10:**
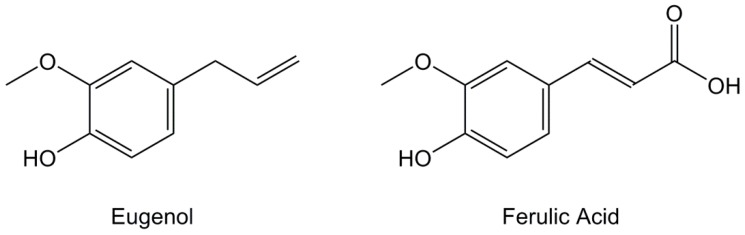
Structures of eugenol and ferulic acid.

Other phenolic compounds can be extracted from various plant essential oils such as 4-allyl-2-methoxyphenol, commonly known as eugenol (Figure 10), obtained from clove oil where it accounts for 80% [119,120,121,122], Jamaican chili, cinnamon and bay leaves from California. It is a renewable resource, but it is also considered safe, non-carcinogenic and non-mutagenic by the U.S. Food and Drug Administration and used as a food flavoring agent. Furthermore, it exhibits several pharmacological properties such as anesthetic, antioxidant, and antimicrobial activities. Apart from extraction, eugenol can be synthesized by allylation of guaiacol, another bio-based phenolic derivative. The main advantage of eugenol is that it exhibits a carbon-carbon double bond with fair reactivity and a phenolic group, thus allowing various functionalizations.

Lignocellulosic biomass may also contain *p*-coumaryl, coniferyl and sinapyl acids, depending on the plant species, that act as crosslinkers between lignin and polysaccharides (cellulose and hemi-cellulose) to increase the rigidity of the materials. Among these, 3-methoxy-4-hydroxycinnamic acid (Figure 10), also known as ferulic acid, is an abundant derivative that can be extracted in good yields from various non-food resources such as bagasse, rice, wheat and sugar beet roots [123,124,125,126] and has both a hydroxyl group and an aliphatic carboxylic acid, thus enabling functionalization. It also exhibits antioxidative, anti-tumor, photoprotective and anti-hypertensive activities.

## 5. Bio-Based Aromatic Epoxy Compounds

### 5.1. Mono-Aromatic Epoxy Compounds

Mono-aromatic glycidyl compounds are defined by having strictly one aromatic ring per molecule and by having two or more epoxy groups per molecule. As previously stated, the characteristic structure of BPA, e.g., its length, its two aromatic rings and alcohols functions greatly influence its endocrine disruptor activity. Thus, mono-aromatic molecules with reduced hydrophobic skeleton may potentially be less likely to bind with estrogen receptor, making their epoxy monomers interesting candidates for BPA substitution, however, specific studies would be necessary before these compounds can be proposed as BPA substitutes.

#### 5.1.1. Phenyl-Based Di-Functional Epoxy Monomers

**Figure 11:**
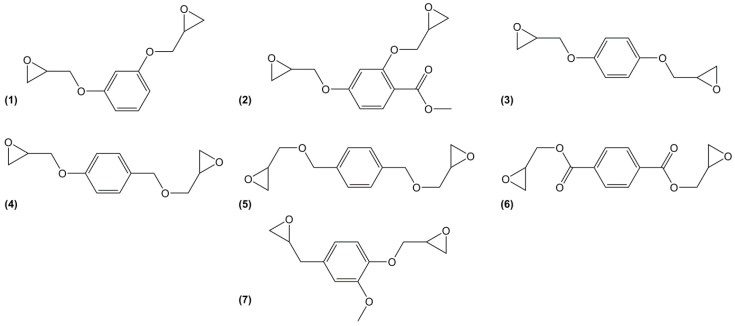
Mono-aromatic phenyl-based di-functional epoxy compounds.

Various mono-aromatic phenyl-based epoxy monomers have been considered as potential candidates for the replacement of DGEBA (Figure 11). One of the simplest, diglycidyl ether of resorcinol (**1**), is obtained from resorcinol, a *meta*-substituted di-phenol that can be obtained from biomass by fermentation [127] of catechins or by fermentation of glucose into inositol, chemical conversion of the latter into 1,3,5-benzenetriol (or phloroglucinol) and finally reduction. As a part of catechin’s skeleton structure, it has been used as model molecule for catechin characterization [128]. It is commercially available or can be prepared by direct *O*-glycidylation of resorcinol with good yield (87%) [38]. Because of the resonance and inductive effects of the *meta*-substituted aromatic ring, diglycidyl ether of resorcinol should be more reactive than its *para*-substituted analogue, diglycidyl ether of hydroquinone (**3**) [129]. Its high toxicity may explain why this epoxy has not been much used as material component. Similarly, a substituted resorcinol, methyl-2,4-dihydroxybenzoate has been glycidylated with epibromohydrin (**2**) with a 78% yield but no materials have been developed from this compound [24]. On the contrary, diglycidyl ether of hydroquinone, the *para* isomer or resorcinol can be obtained via the microbial synthesis of phloroglucinol or quinic acid from glucose, converted into hydroquinone (or resorcinol) [130]. This epoxy was cured with diethyl toluene diamine (EPIKURE W) and the obtained materials exhibited slightly higher glass transition temperature (*T*_g_) than those based on DGEBA (up to 8 °C) [131], being the greatest data obtained with a di-functional epoxy. However, contrary to resorcinol, hydroquinone is carcinogen (H351) and mutagen (H341).

The same research team prepared the diglycidyl ether of *p*-xylene alcohol (**5**) by direct glycidylation of the non-toxic 1,4-benzenedimethanol with an overall yield of 90% and a purity of 99% assessed by ^1^H NMR [131]. It has been cured with cycloaliphatic and aromatic diamines, 4,4′-methylene biscyclohexylamine (PACM) and diethyl toluene diamine (EPIKURE W), respectively yielding materials with *T*_g_ values up to 67 °C lower than those of DGEBA-based equivalents, because of the methylene linkages.

The ester form of the latter compound was prepared by direct glycidylation of the non-toxic terephthalic acid [132], to form diglycidyl ester of terephthalic acid (**6**) with a yield of 80%. This epoxy was cured with methylhexahydrophthalic anhydride and poly(propylene glycol) bis(2-aminopropyl ether). The materials showed similar *T*_g_ values as those based on DGEBA with both curing agents. Furthermore, terephthalic acid is synthesized from *p*-xylene that can be obtained from bio-mass by various processes [133]. Due to the large amount of by-products using both direct glycidylation and allylation methods, You et al. [134] prepared diglycidyl ester of terephthalic acid by esterification of diacyl chloride by glycidol, with a 50% yield. Diglycidyl ester of terephthalic acid was cured with carboxylic acid derivatives, and the corresponding materials were used as biocompatible, biodegradable and functional polymers for biomedical applications. No thermal properties were reported. However, the low hydrolytic stability of aromatic ester groups is expected to negatively impact the potential materials [134].

The double functionalization of eugenol (**7**) requires a three-step route: (i) protection of the hydroxy group via an acetylation using acetic anhydride; (ii) oxidation of the double bond using *m*CPBA and (iii) glycidylation by deacetylation using epichlorohydrin. The resulting solid was obtained with an overall yield of 53% [119]. The epoxy was cured with anhydrides and the obtained materials showed similar thermal and mechanical properties than those of DGEBA equivalents. For example, when diglycidyl ether of eugenol was cured with hexahydrophtalic anhydride, the obtained *T*_g_ reached 114 °C, which was slightly higher than that of DGEBA counterparts (106 °C).

**Figure 12:**
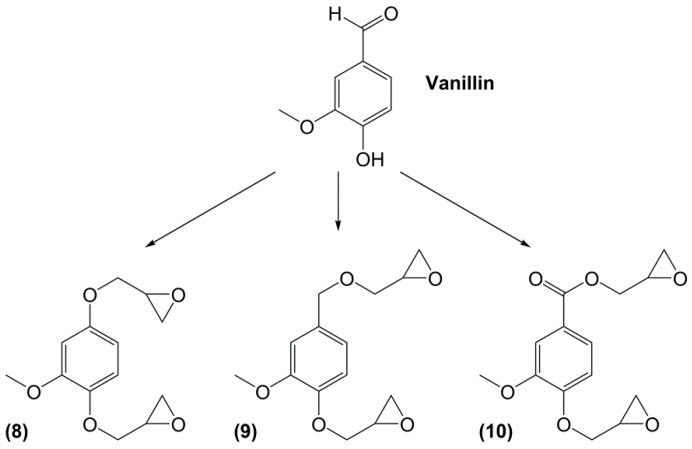
Diglycidyl ether compounds obtained from vanillin: (**8**) diglycidyl ether of vanillyl alcohol, (**9**) diglycidyl ether of methoxyhydroquinone and (**10**) diglycidyl ether of vanillic acid [135].

Eugenol can be used as a precursor for vanillin synthesis. The latter is an important bio-based phenolic aldehyde, which is the main component of vanilla bean extract, widely used as flavouring in food, beverages and pharmaceuticals [135]. It is also a bio-sourced chemical derived from lignin currently produced by Borregaard [123] that easily allows to lead to diepoxy compounds, the structures of which are showed in Figure 12. All diglycidyl ethers deriving from vanillin can be prepared by direct *O*-glycidylation of the corresponding alcohol derivatives, with good yield (85%–89%) [27]. It is interesting to note that the alcohol derivatives, e.g., 4-hydroxy-3-methoxybenzyl alcohol, 2-methoxyhydroquinone and 4-hydroxy-3-methoxybenzoic acid are all commercially available, non-toxic and also used for food flavouring. The three diglycidyl ether compounds were cured with isophorone diamine (IPDA) and showed *T*_g_ values lower than that of DGEBA by 69 (**9**), 34 (**8**) and 14 °C (**10**). The authors considered that the presence of a methylene spacer decreased *T*_g_ by 35 °C and when carbonyl group was used as spacer, *T*_g_ increased by 20 °C. Additionally, when diglycidyl ether of hydroquinone and diglycidyl ether of methoxyhydroquinone were compared, the presence of the methoxy group involved a decrease of *T*_g_ by 25 °C. Similarly, when comparing (**8**) and diglycidyl ether of eugenol (**7**), it seems that the loss of the (CH_2_-*O*-) group between the oxirane ring and the aromatic ring allows to reach higher glass transition temperatures, closer to those of DGEBA-based materials.

Recently, Hernandez et al. [17] continued the researches to determine the influence of the methoxy group of diglycidyl ether of vanillyl alcohol (DGEVA) (**9**) by comparing it with diglycidyl ether of gastrodigenin (DGEGD) (**4**). When cured with 4,4′-methylbiscyclohexylamine, the DGEVA-based materials exhibited a lower *T*_g_ than the DGEGD ones due to the methoxy moiety, but it appears that it enables to obtain a higher rigidity in the glassy state, as observed via the higher storage modulus.

**Scheme 8:**
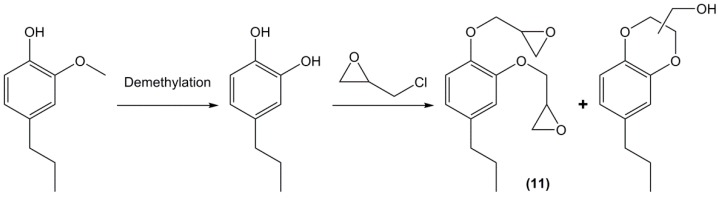
Synthetic pathway to glycidylated propylcatechol as described by Zhao and Abu-Omar [138].

Following its previous work on lignin depolymerisation using hydrogenation in the presence of Zn/Pd catalysts [136,137] the team of Abu-Omar [138] synthesized di-epoxides from propylcatechol (**11**) (Scheme 8). As previously explained, the presence of hydroxyl groups in *ortho* position will yield benzodioxane derivatives during the glycidylation step, but the authors claimed to have improved the synthesis conditions in terms of epoxy equivalent weight and mass ratio. The epoxy monomer was then mixed with octadecylamine-modified nano-montmorillonite and cured with diethyl triamine. The bio-based polymers were characterized in terms of thermal stability and mechanical properties but no possible leaching of the benzodioxane derivatives non-integrated in the network was discussed.

**Figure 13:**
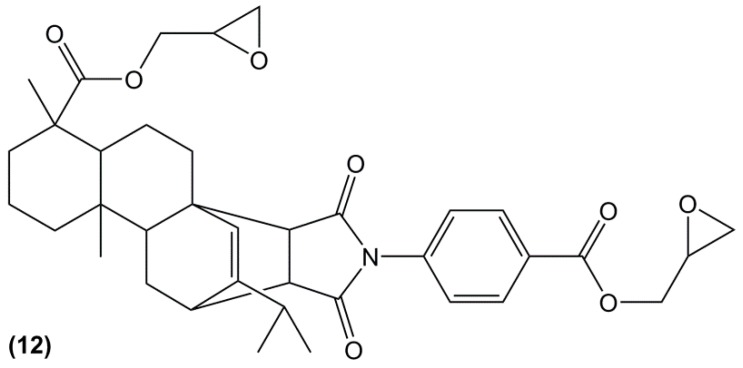
Chemical structure of a rosin-based diepoxy compound [139].

Another abundant and natural product is rosin. With a production of approximately 1.2 million of tons per year [19], this compound is obtained by heating fresh tree resin to remove the volatile liquid terpenes. It thus contains a mixture of isomerized acid with large hydrogenated phenanthrene ring structures providing them with high rigidity. Most of them do not contain aromatic structures, but Liu et Zhang [139] recently synthesized a mono-aromatic di-glycidyl ester based on rosin acid (**12**) (Figure 13), cured it with 1,2-cyclohexanedicarboxylic anhydride and compared the results with DER332, an epoxy resin from Dow Chemical Company cured in the same conditions. The resulting material exhibited a *T*_g_ of 154 °C slightly higher than its counterpart, and a good thermal stability with a temperature of 5% weight loss of 311 °C.

#### 5.1.2. Phenyl-Based Poly-Functional Epoxy Monomers

**Figure 14:**
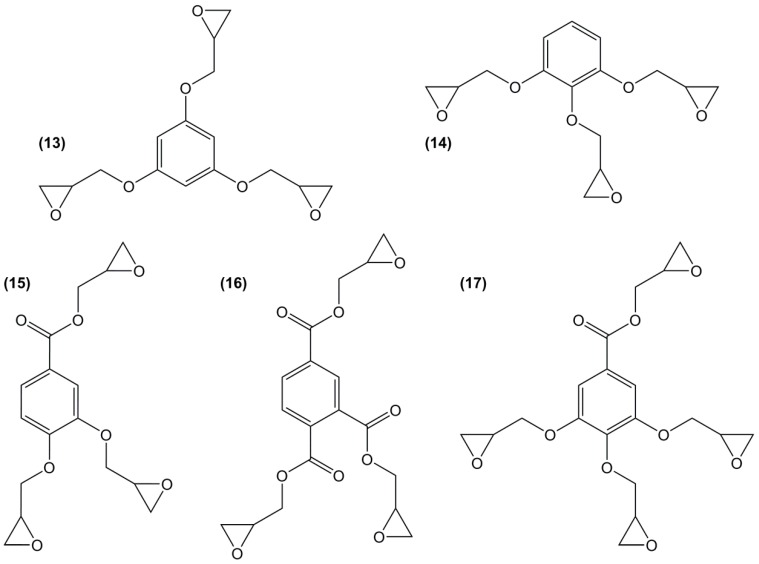
Mono-aromatic phenyl-based poly-functional epoxy compounds.

Phloroglucinol is a bio-based tri-phenol and is used as an active ingredient in medicines (SPASFON). The glycidylated compound (**13**) (Figure 14) was prepared by direct glycidylation with a yield of 68%, leading to a mixture mainly containing triglycidyl ether of phloroglucinol [140]. Its symmetric tridimensional and tri-functional *meta*-substituted structure makes it the most reactive epoxy [129]. The reached materials exhibited much higher *T*_g_ than those of DGEBA, up to 60 °C for linear aliphatic [141] and 20 °C for cycloaliphatic diamines [142].

Another simple tri-functional glycidyl ether is based on pyrogallol (**14**), which is also a bio-based molecule obtained from decarboxylation of gallic acid extracted from hydrolysable tannins. Unfortunately, it is mutagen (H341) and its structure produces more bulkiness than its *meta*-substituted analogue. Moreover, as previously described, the *ortho* position of the phenol groups yields a 50:50 mixture of triglycidyl ether of pyrogallol and benzodioxane derivatives, with an overall yield of 66% [38]. The low yield and by-products synthesis were likely the reason why no material has been prepared from this epoxy.

Similarly, protocatechuic acid, which is a relatively toxic (H315, H319, H335) major metabolite of antioxidant and anti-inflammatory bio-based polyol found in green tea, yields a mixture of 60% of the triglycidyl ether (**15**) and 9% of the benzodioxane derivative [38]. The presence of the carboxyl group in meta-position apparently limited the cyclization compared to pyrogallol. Despite the good yield, no material has been reported.

Another triglycidylated compound was synthesized from trimellitic acid (**16**), which is a relatively toxic triacid. The epoxy form was found at 25% in a mixture with 75% of diglycidyl terephthalate acid ester, supplied by Vantico-Switzerland. This mixture was used as polyester curing agent for coating applications. No thermal properties of the intrinsic materials have been reported [143,144], but a low hydrolytic stability is expected similarly to the diglycidyl ester of terephthalic acid (**6**).

Gallic acid is a bio-based, trihydroxybenzoic acid compound encountered in the plant hydrolysable tannins, as gallic acid derivatives (esters, glycosides) or as the acyl group of some polyols (glucose, quinic acid). It is as toxic as protocatechuic acid and is the only mono-aromatic compound with four functions that can be glycidylated. Tetraglycidyl ether of gallic acid (**17**) can be prepared by direct glycidylation or allylation [22,145]. The direct glycidylation of gallic acid allowed to reach the targeted product with a yield of 68% [22,146,147], whereas, the allylation route led to a better control of the functionalization, but a lower overall yield of tetraglycidyl derivative of 48%. The materials cured using isophorone diamine reached *T*_g_ values of 73 °C higher than that of DGEBA, which is the highest value obtained for mono-aromatic epoxy compounds [22].

Cardanol has also been considered as a BPA substitute for epoxy monomer synthesis. The conversion of the C=C double bonds to epoxide groups can be almost complete by reaction with hydrogen peroxide in the presence of formic acid and *p*-toluenesulfonic acid, with a yield of 82%. The resulting product is only glycidylated along the alkyl chain and is used for its antioxidative activity in soybean oil [106]. 

**Figure 15:**
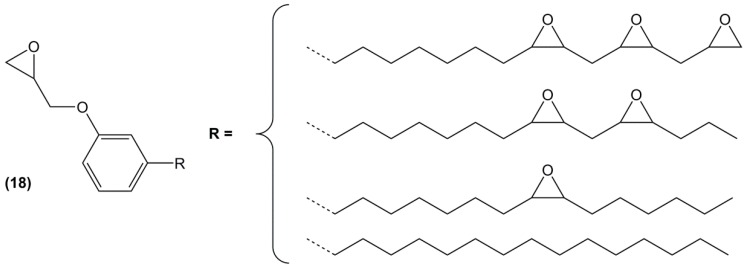
Theoretical structure of epoxidized cardanol.

Fully epoxidized cardanol (**18**) (Figure 15) was prepared by direct *O*-glycidylation of the phenol, followed by double bonds oxidation using *m*CPBA (78%) or perbenzoic acid and used as a natural plasticizer for PVC films [105] or as diluent to improve the mechanical properties of DGEBA cured by phthalic anhydride for glass-fiber reinforcement applications. For this latter application, *T*_g_ increased with the amount of epoxidized cardanol, from 145 to 180 °C using 40% of diepoxy cardanol [107]. No epoxy/amine-based material has been reported, likely because the epoxy groups along the alkyl chain exhibit a lower reactivity toward amines. In fact, glycidyl ether compounds are found to be the most reactive species towards amine compounds because of the inductive effect of the oxygen on the epoxy-ring and the fact that the ether oxygen is able to form hydrogen bonds with the amine [129]. An interesting approach is thus to combine both characteristics in one molecule.

**Figure 16:**
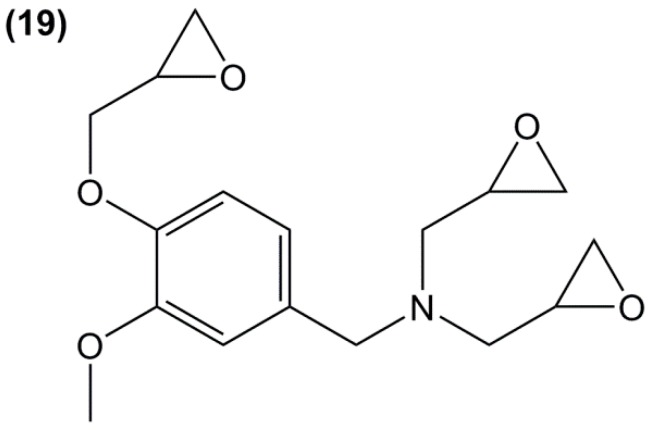
Aromatic derivatives with epoxy groups grafted on nitrogen atoms.

Triglycidyl ether of vanillylamine (**19**) (Figure 16) is the only tri-functional mono-phenolic glycidylated derivative synthesized from the bio-based and non-toxic vanillin reported in the literature [148]. It was prepared in a four-step synthesis from hydroxylamine hydrochloride: (i) aldoximation of vanillin; (ii) reduction of the aldoxime using dihydrogen in the presence of Pd/C; (iii) neutralization in basic medium leading to a potential alkanolamine hardener; (iv) direct *N*-glycidylation of the resulting vanillylamine, with an overall yield of 61%. The materials cured with isophorone diamine showed similar *T*_g_ than that of triglycidyl ether of phloroglucinol.

#### 5.1.3. Epoxy Monomers Based on Other Aromatic Rings

**Figure 17:**
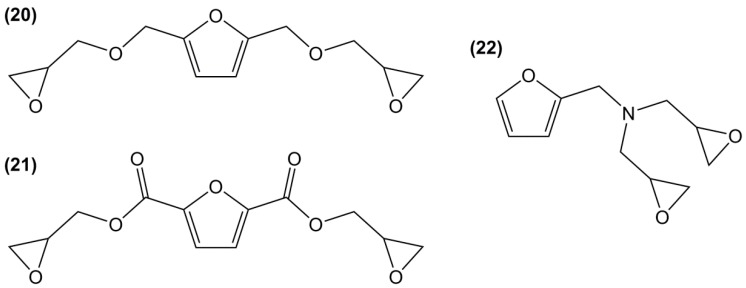
Furan-based diglycidylated compounds.

Furfuryl diglycidyl ether (**20**) (Figure 17) was prepared by direct glycidylation of the 5-hydroxymethyl-2-furfural, with a yield of 60% and a purity of 99%—however 5-hydroxymethyl-2-furfural is a toxic substance. Hu et al. [149] found that the overall reactivity of furan derivatives was significantly greater than that of the phenyl analogue and led to materials with a glass transition temperature higher than their benzene analogs. However, in a recently corrected article [150], the research group changed their observations and conclusions as the benzyl-based materials showed higher *T*_g_ values than the furan-based ones, due to a higher freedom degree of the furan ring and the hydrogen bonding between atoms in the furan rings and hydroxyl groups created by the epoxy opening.

In another article, their further investigation on furfuryl diglycidyl ether cured with difurfurylamine led them to conclude that each methylene spacer per ring reduced the *T*_g_ by 32–34 °C and that furanyl structure led to higher freedom degree, which decreased *T*_g_ [151]. When compared with those obtained from DGEBA, the mono-aromatic furan-based materials showed a *T*_g_ in between 66 and 97 °C lower.

Furfuryl diglycidyl ester (**21**) was prepared by allylation and epoxidation of the bio-based but irritant 2,5-furandicarboxylic acid, in an overall yield of 59% [132]. The authors reported that the furandicarboxylic-based materials displayed higher curing reactivity, higher *T*_g_, similar mechanical properties and thermal stability than those based on terephtalic derivatives. The freedom degree of furandicarboxylic structure is lower than with a methylene spacer, which is consistent with previous observations [135,151], but lower stability toward hydrolysis is expected.

*N*,*N*′-Diglycidyl furfurylamine (**22**) was synthesized by double *N*-glycidylation of the relatively toxic 2-aminomethylfurfuran, with an overall yield of 50% [152]. When the resulting multi-functional epoxy was cured with maleimide and anhydride species, stable thermally reversible linkages were formed, leading to self-healing thermosetting materials.

**Figure 18:**
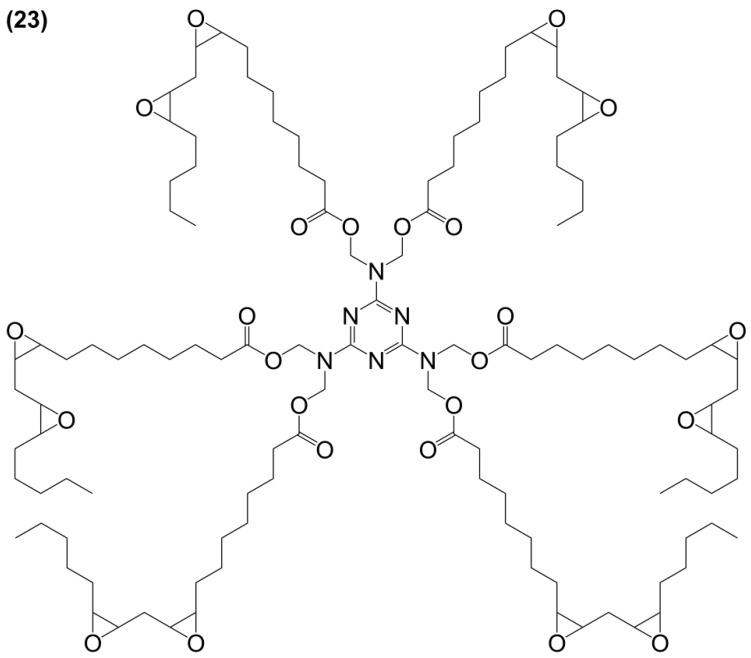
Structure of the glycidylated hexa(linoleoyl hydroxymethyl) melamine [153].

Recently Liu et al. [153] developed a six-armed epoxy resin (**23**) based on linoleic acid and hexamethylol melamine in a two-step process of esterification and epoxidation with H_2_O_2_ (Figure 18). Their goal was to reduce the high flexibility induced by the long aliphatic chain of the vegetable oils by introducing a rigid aromatic group. Resins were cured using 4-methyl hexahydrophthalic anhydride and 1,8-diazabicyclo[5.4.0]undec-7-ene as a catalyst and exhibited high degrees of cure, *T*_g_ values between 28 and 42 °C higher than those observed when epoxidized sucrose soyate was used. However, their temperature at 5% weight loss turned out to be from 20–90 °C lower, thus indicating a poorer thermo-oxidative stability. With similar hardener content, the resin presented a bio-based content of 70% vs. 80% for the sucrose soyate-based one.

#### 5.1.4. Conclusions

Bio-based mono-aromatic epoxy materials show interesting properties in terms of *T*_g_ and thermal stability. While cardanol-based materials exhibit flexibility and low *T*_g_ values, tri- and tetraglycidyl ether, led to materials with higher *T*_g_ than DGEBA-based materials. Among the di-functional mono-aromatic substances, only the materials based on epoxy with no spacer and separated by a carbonyl group showed higher *T*_g_. Unfortunately, since most of these epoxides are in development, their toxicity is not known and at that moment, little information is available on the potential endocrine disruption of their precursors. Table 1 and Table 2 gather the known data on materials based-on mono-aromatic di- and poly-epoxy monomers from renewable resources.

### 5.2. Di-Aromatic Epoxy Compounds

Its bisphenolic structure provides BPA with high thermo-mechanical properties but also toxicity. In an attempt to mimic but alter the BPA structure to match its qualities but not its defaults, researchers have developed various di-aromatic derivatives based on bio-resources. The diaromatic glycidyl ether compounds are defined as having strictly two benzene rings bearing two or more epoxy groups per molecule. As the spacer between these two aromatic rings plays an important role in the estrogenic disruption of BPA, the epoxy monomers will be presented in the following sections according to the number of atoms between the two aromatic moieties: (i) two benzene rings without spacer; (ii) separated by one atom; (iii) separated by two atoms and (iv) separated by long spacers.

#### 5.2.1. Two Aromatic Rings without Spacer

**Scheme 9:**
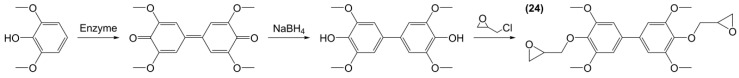
Synthetic pathway developed by Grelier et al. [155] to obtain diphenolic derivatives from 2,6-dimethoxyphenol.

Grelier et al. [155] synthesized a diglycidyl ether derivative (**24**) from the bio-based but highly toxic for the environment (H411) 2,6-dimethoxyphenol using a three-steps pathway: (i) enzymatic coupling using laccase from *Tramates versicolor*; (ii) reduction of the carbon-oxygen double bonds into alcohols and (iii) *O*-glycidylation with epichlorohydrin (Scheme 9). The di-epoxy monomer obtained was crosslinked using isophorone diamine yielding a material that exhibits a *T*_g_ of 126 °C and a good thermal stability with a *T*_d,5%_ of 312 °C. Unfortunately, no DGEBA comparison was provided.

**Scheme 10:**
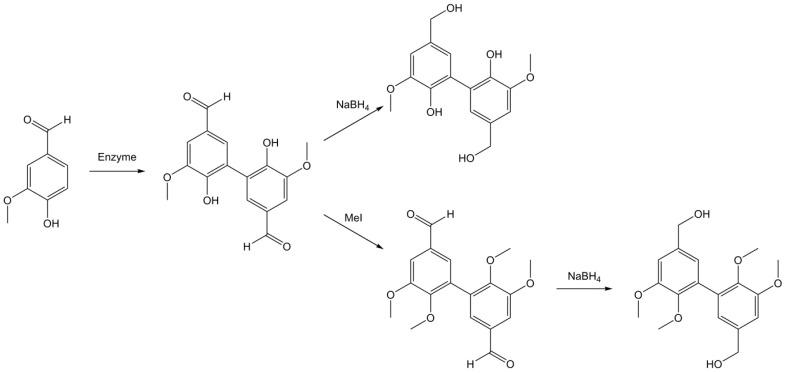
Synthetic pathway developed by Grelier et al. [156] to obtain diphenolic derivatives from vanillin.

Following this method, the same team synthesized di-phenols from vanillin [156] (Scheme 10). A methylation step using methyl iodide can be applied prior to the reduction in order to obtain di-functional derivatives. No epoxides were synthesized from these compounds but a simple glycidylation step could easily lead to potential bio-based monomers.

#### 5.2.2. Two Aromatic Rings Separated by One Atom

**Figure 19:**
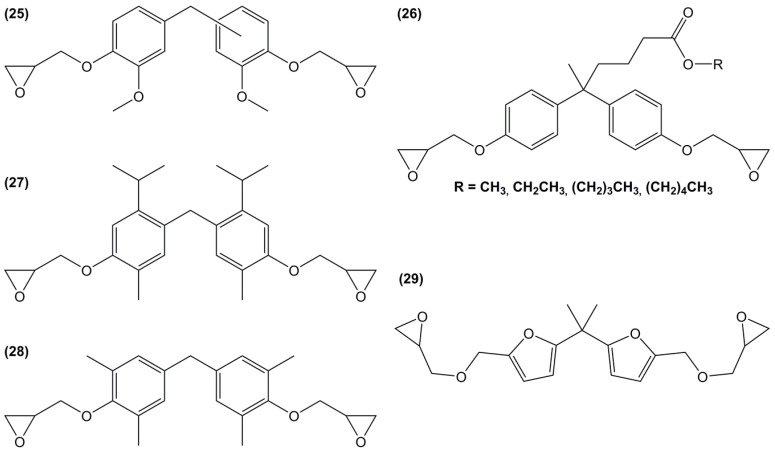
Bio-based di-aromatic epoxy derivatives with a single carbon atom spacer.

Hernandez et al. [17] recently synthesized epoxy resins by a two-steps pathway involving the electrophilic condensation of vanillyl alcohol and guaiacol, a mono phenolic derivative that can be obtained in large quantities when carrying out the pyrolysis of lignin. This original method is an interesting alternative to the use of formaldehyde, especially for the synthesis of an analogue to bisphenol F. The obtain bis-guaiacol was then glycidylated (**25**) (Figure 19) and cured with 4,4′-methylene-biscyclohexylamine (Amicure PACM), yielding a material with a *T*_g_ value of 111 °C compared to 158 °C for the DGEBA-based material and a slightly lower thermal stability.

*N*-alkyl diphenolate diglycidyl ethers (**26**) were synthesized by Maiorana et al. [157] in yields ranging from 85% to 97% in a two-step pathway using diphenolic acid. First, it was esterified with various alcohols and then glycidylated using epichlorohydrin. The advantage of these epoxides is their liquid state at room temperature and the possibility to have fully bio-based materials. In fact, levulinic acid is produced from cellulose by the acid hydrolysis of C_6_ sugars at the pilot plant scale by Biofine [110] and Segetis [158]. The materials cured with isophorone diamine showed glass transition temperatures in between 86 and 158 °C, depending of the alkyl chain, which is lower than that of DGEBA-based equivalents (165 °C). *T*_g_ decreases with increasing alkyl chain length. No trend was observed regarding storage modulus or tensile strength but materials exhibited mechanical properties similar to DGEBA-based materials and slightly lower thermal stability.

Harvey et al. [117] synthesized di-epoxides (**27**) through methylene bridging between two carvacrol molecules with 1,3,5-trioxane in dilute HCl at elevated temperature (Figure 19) and *O*-glycidylation using epichlorohydrin, with an overall yield of 42%. The resulting monomer was cured with 4,4′-diaminodiphenyl methane and 4,4′-methylene bis(5-isopropyl-2-methylaniline), also prepared from *p*-cymene [116]. The *ortho*-methylene substituents led to lower degree of cure and higher moisture resistance, and likely lower hydrolysis. The obtained materials showed glass transition temperatures ranging from 143 to 161 °C, which is slightly lower than DGEBA-based materials. When this di-epoxide was cured with 4,4′-diaminodiphenyl methane and compared with diglycidyl ether of tetramethylbisphenol F, the material based on the former showed lower *T*_g_ than the latter, up to 23 °C.

Diglycidyl ether of tetramethylbisphenol F (**28**) was prepared in a two-step synthesis route from 2,6-dimethylphenol and the mutagenic and carcinogenic (H341-350) formaldehyde: (i) aldol condensation reaction between formaldehyde and two molecules of 2,6-dimethylphenol followed by (ii) the direct *O*-glycidylation using epichlorohydrin, with an overall yield of 79% [159]; The *T*_g_ of the material cured with 4,4′-diaminodiphenyl methane showed a high *T*_g_ value, up to 184 °C. When this epoxide was compared with its non-spacer analogue, 3,3′,5,5′-tetramethyl-4,4′-biphenol, the additional methylene spacer led to a slight decrease of the glass transition temperature, up to 15 °C lower.

Diglycidyl ether of bisfuran (**29**) was prepared in a four-step synthesis from the bio-based 2-furoic acid and acetone: (i) protection of the carboxylic groups using methanol; (ii) coupling of two resulting molecules in the presence of acetone and concentrated sulfuric acid; (iii) reduction of the ester groups using lithium aluminum hydride and then; (iv) direct *O*-glycidylation of the di-alcohol, with an overall yield of 48% [113]. However, no materials have been prepared from this epoxy resin.

**Figure 20:**
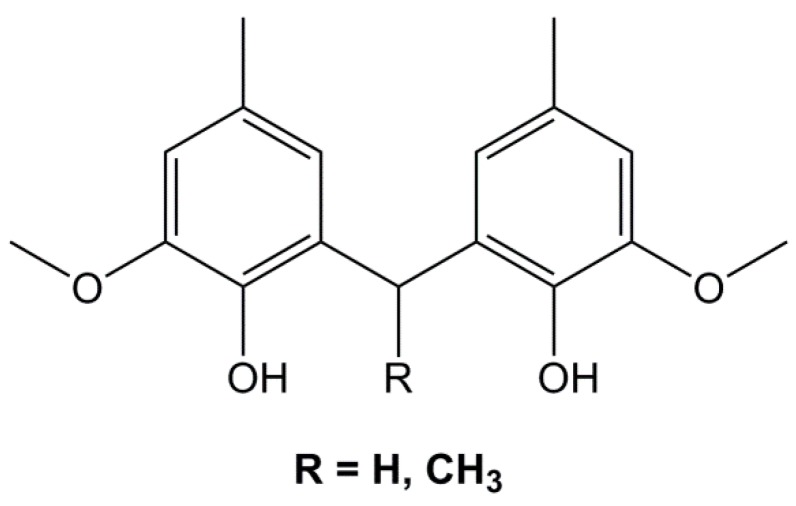
Creosol-based di-phenolic derivative synthesized by Meylemans et al. [160].

Meylemans et al. [160] proposed an interesting strategy to synthesize di-phenols from creosol, a phenolic derivative coming from the reduction of vanillin (Figure 20). Through Zn(AcO)_2_ or acid catalyzed coupling with aldehydes, they obtained two different di-phenol suitable for epoxide synthesis. Unfortunately, no glycidylation step was carried out on these original compounds, although the use of bio-based aldehydes such as benzaldehyde may produce highly bio-sourced monomers as well as greatly influence the mechanical and thermal properties. However, despite the presence of methoxy moieties and a *meta* substitution, the di-phenol derivatives exhibit a structure very close to bisphenol E and F, according to the aldehyde chosen, thus requiring toxicological studies to determine their potential dangerousness or estrogen disruptor behavior.

#### 5.2.3. Two Aromatic Rings Separated by Two Atoms

**Figure 21:**
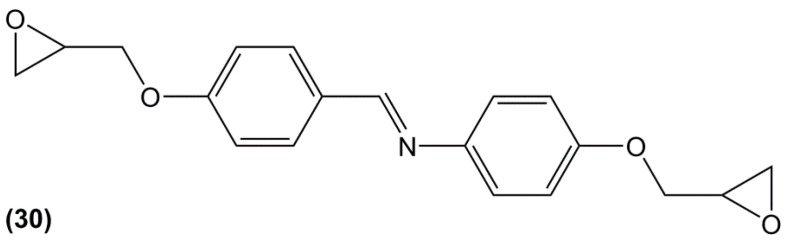
Epoxy resin based on *p*-aminophenol and *p*-hydroxybenzaldehyde synthesized by Duann et al. [161].

Duann et al. [161] prepared a bis-phenolic glycidylated derivative by reacting *p*-aminophenol and *p*-hydroxybenzaldehyde prior to a direct epoxidation with epichlorohydrin (**30**) (Figure 21). *p*-hydroxybenzaldehyde can be obtained from biomass by depolymerizing lignin [162] but to our knowledge no bio-based commercial *p*-aminophenol is currently available. However, Ng et al. [163] claimed in a patent to have developed a process for producing this compound using genetically engineered microorganisms and biomass-based sugars, possibly providing this resin with a high renewability percentage. After curing with 4,4′-diamino diphenyl methane, they obtained a material with a *T*_g_ of 200 °C and a temperature of 5% weight loss under air of 342 °C. Unfortunately, no comparison with a DGEBA-based material was provided to determine the role of the carbon-nitrogen double-bond on the thermal and mechanical properties.

**Scheme 11:**
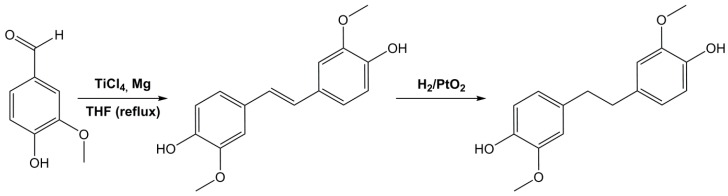
Synthesis of di-phenolic compounds from vanillin according to Harvey et al. [164].

Using vanillin as a starting material, Harvey et al. [164] synthesized a di-phenol stilbene using titanium chloride and magnesium, and further hydrogenated it on platinum oxide (Scheme 11). These compounds were turned into cyanates and used to obtain polycyanurates, but no glycidylation step has been considered by the authors despite the interesting potential of these molecules for di-epoxy monomers.

#### 5.2.4. Two Aromatic Rings Separated by More Than Two Atoms

The small size of BPA allows it to mimic the natural estrogen 17β-estradiol and enter the estrogen receptor pocket. Thus, by increasing the length of the spacer, it may be possible to decrease the activity of the epoxy monomer precursors. In this part, all monomers with long aliphatic or cycloaliphatic spacer will be presented, including both di- and poly-epoxy monomers.

##### Di-Epoxy Monomers

**Figure 22:**
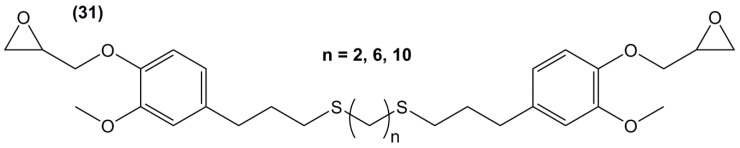
Eugenol-based epoxy prepolymers synthesized by Zou et al. [165].

Recently, Zou et al. [165] synthesized bio-based epoxy monomers by coupling glycidylated eugenol via photo-initiated thiol-ene reaction using three different aliphatic di-thiols (**31**) (Figure 22). The resins were cured by 4,4′-diaminodiphenylmethane and the resulting materials exhibited *T*_g_ and *T*_α_ values ranging from 39 to 60 °C by DSC and 54 to 70 °C by DMA, respectively. As expected, the glass transition decreased when increasing the di-thiol chain length.

**Scheme 12:**
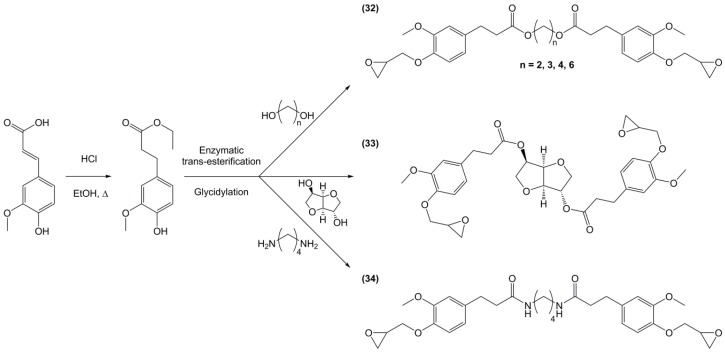
Synthetic pathway to obtain bio-based di-epoxy monomers from ferulic acid proposed by Maiorana et al. [123] and Ménard et al. [166].

Following the same synthetic pathway, Maiorana et al. [123] and Menard et al. [166] synthesized bio-based bis-ferulate epoxy monomers (**32**) (**33**) and (**34**) (Scheme 12) via a three step synthetic pathway: (i) synthesis of ethyl ferulate by reacting with HCl and hydrogenating over Pd/C; (ii) enzyme-catalyzed transesterification with various bio-based diols including *n*-alkyl diols and isosorbide or transamidification with 1,4-diaminobutane; and (iii) *O*-glycidylation with epichlorohydrin in high yields. The epoxy monomers were cured by isophorone diamine, 1,10-diaminodecane and difurfurylamine. Regarding the thermosets based on the *n*-alkyl bis-ferulates and IPDA, the *T*_α_ values ranged from 50 to 65 °C and decreased while increasing the diol chain length, compared to 170 °C for DGEBA-based material. The change of ester to amide bonds lead to higher *T*_g_ values thanks to the hydrogen bonding induced by the nitrogen atoms. These values were roughly similar to those obtained with the isosorbide derivative, the cycloaliphatic structure of which brought more rigidity to the polymeric network. However, none of these materials could compete with DGEBA-based resources in terms of *T*_g_/*T*_α_. An interesting point to mention nonetheless is that Maiorana et al. [123] also focused on the degradability of the obtained materials for thermosets recyclability issues, and studied the estrogenic activity of bisferulates compared to bisphenol A and 17β-estradiol. The *n*-alkyl bis-ferulates derivatives showed no significant estrogenic activity for ER α at concentrations where bisphenol A does, highlighting the potential of these compounds as a safer substitute for BPA.

**Figure 23:**
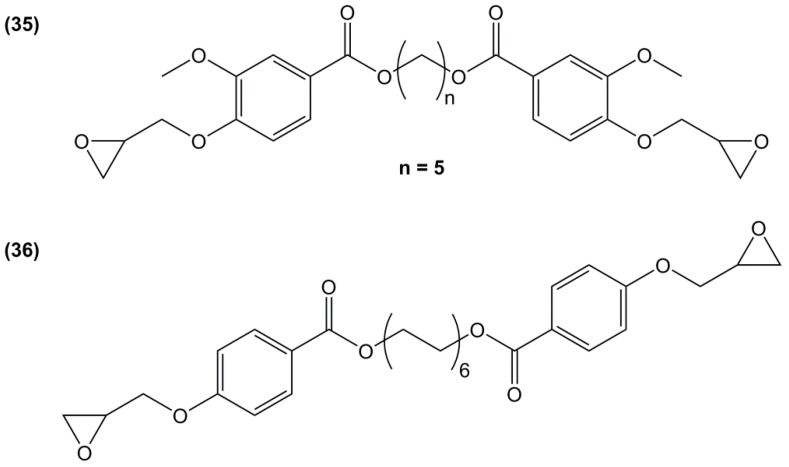
Di-epoxy monomers with longer ester spacers between the aromatic rings.

In a similar way, Aouf et al. [40] synthesized glycidylated bio-based bis-vanillic acid (**35**) in a three-steps pathway: (i) synthesis of bis-vanillic acid by reaction with 1,5-dibromopentane; (ii) allylation of the hydroxyl functions with allyl bromide and (iii) epoxidation of the allyl bond with an enzymatic catalyst (Figure 23). Unfortunately, no materials were synthesized with these di-epoxy monomers.

Fourcade et al. [167] used 1,6-hexanediol to synthesize an epoxy resin using a two-steps synthetic route: (i) phenolization of the 1,6-hexanediol using ethyl-4-hydroxybenzoate with 92% of yield; and (ii) direct *O*-glycidylation using epichlorohydrin in the presence of sodium carbonate (**36**) (Figure 23). The resulting epoxide was used as a DGEBA diluent and blended with 50%–90% of DGEBA and then cured with dicyandiamide. The glass transition temperature of the blend materials ranged from 77 to 109 °C, decreasing when increasing the amount of aliphatic epoxide. Materials exhibited high thermal stabilities under air, but unfortunately no sample without DGEBA was mentioned. While the 1,6-hexanediol used by the authors was oil-based, it can be obtained by hydrogenation of adipic acid coming from different biomass such as glucose, lignin and fatty acids [168]. It is considered lowly toxic [169] and is not labelled as carcinogenic or mutagen. Ethyl-4-hydroxybenzoate is obtained by esterification of 4-hydroxybenzoic acid, a naturally occurring product synthesized industrially from phenol, carbon dioxide and bio-ethanol. Moreover, the authors used epichlorohydrin from glycerol (Epicerol^®^ process) making the resins possibly highly renewable, although no value was given.

**Figure 24:**
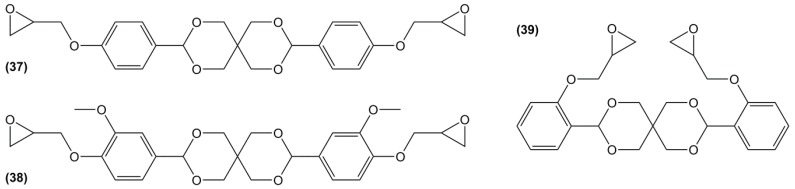
Diglycidyl ether derivatives containing spiro rings.

Diglycidyl ether derivatives including spiro-ring as spacer can be prepared in a two-step pathway from non-toxic, bio-based reactants, e.g., vanillin [27,135,170,171] and pentaerythritol [167]: (i) condensation of both starting substances and (ii) direct *O*-glycidylation using epichlorohydrin in the presence of sodium hydroxide. However, no other detail, such as reaction yield could be found [172]. In 1986 and 1987, Ochi et al. [173] investigated a series of diglycidyl ether containing spiro-ring building blocks (**37**) (**38**) (**39**), as shown in Figure 24. Materials cured with hexahydrophthalic anhydride showed similar or higher glass transition temperatures than that of their DGEBA counterparts. Curing with more rigid anhydrides, e.g., phthalic anhydride and nadic anhydride allowed to slightly increase *T*_g_, in comparison to hexahydrophthalic anhydride.

**Scheme 13:**
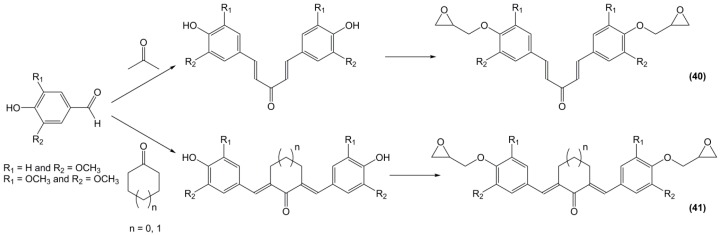
Synthesis of di-epoxy monomers from vanillin and syringaldehyde with acetone or cycloaliphatic ketones according to Rao and Samui [174].

Rao and Samui [174] synthesized diepoxy monomers including bisbenzylidene segments by reaction between vanillin or syringaldehyde with acetone (**40**) and cycloaliphatic ketones (**41**) followed by glycidylation with epichlorohydrin (Scheme 13). These monomers were used to synthesize linear polyesters for photoactive liquid crystal applications but also reacted with benzene-1,3,5-tricarboxylic acid to obtain dendrimers, although their protocol seems more likely to yield crosslinked materials. No data on potential thermo-mechanical properties is thus mentioned.

##### Poly-Epoxy Monomers

**Figure 25:**
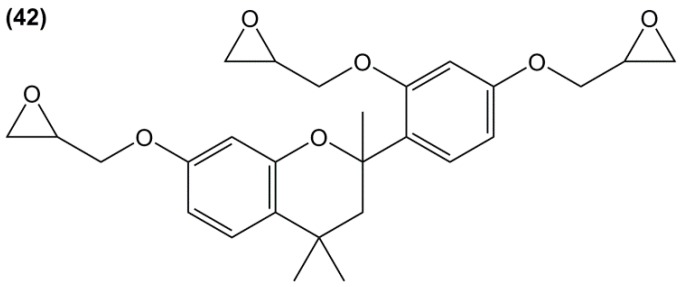
Triglycidyl ether derivative based on resorcinol and acetone.

Triglycidyl ether of polyphenol (**42**) (Figure 25) was synthesized in a two-steps pathway from resorcinol and acetone: a coupling reaction followed by direct *O*-glycidylation using epichlorohydrin [175]. The glass transition temperature of the resulting material cured with diaminodiphenyl sulfone was not observed neither in DSC, nor in DMA, up to 300 °C. The authors concluded that the *T*_g_ was above 300 °C, thus making it much higher than that of DGEBA-based material. The three epoxy groups and the rigidity of the cyclic backbone may in fact explain such a result.

**Figure 26:**
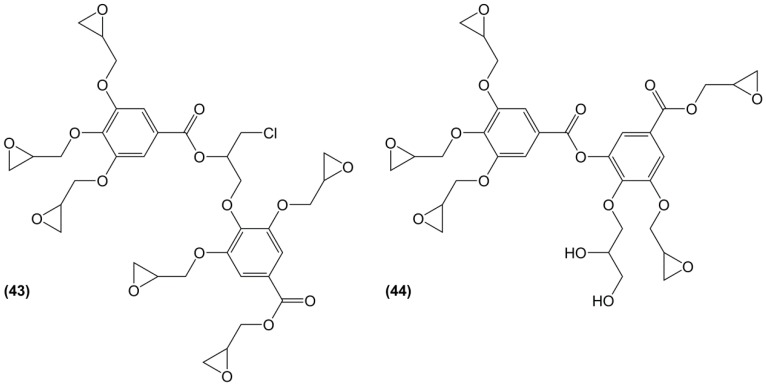
Main oligomers obtained from the glycidylation of depolymerized tara tannins by Aouf et al. [176].

Aouf et al. [176] studied hydrolysable tara tannins as potential candidates for a source of phenolic moieties. These galloylquinic acid oligomers were first depolymerized by tannase-assisted hydrolysis to produce mainly gallic acid and galloylquinic acid. The epoxidation of this mixture was carried out by direct *O*-glycidylation, leading to galloylquinic esters and glycidylated dimerized gallic moieties (Figure 26). The obtained monomer mixture was cured using isophorone diamine and the resulting material showed a *T*_g_ up to 129 °C, which is 20 °C lower than that of DGEBA-based materials in the same conditions. The presence of esters bonds may however make these thermosets more sensitive to hydrolysis and favour the use of condensed instead of hydrolysable tannins.

**Figure 27:**
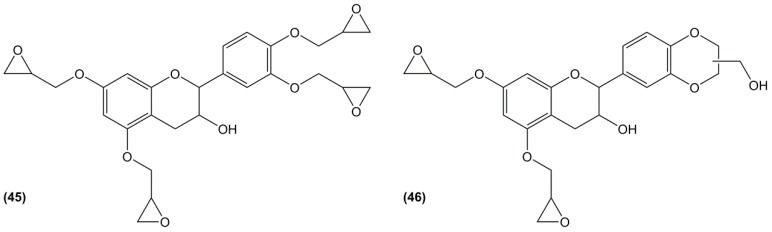
Epoxy monomers obtained by glycidylation of catechin according to Nouailhas et al. [128].

For this reason, Nouailhas et al. [128,177] focused their research on catechin, one of the constitutive units of condensed tannins. Although it is not yet bio-based, it can be obtained by acid-depolymerization as previously described (Section 4.2.). The direct *O*-glycidylation of catechin (Figure 27) led mainly to the tetraglycidylated catechin (**45**) (46%) and to two di-glycidylated benzodioxane derivatives (**46**), with a yield of 18% and 15%, respectively. As the mixture was a solid at room temperature, it was mixed with 25 and 50% of DGEBA as a reactive diluent and cured with Epamine PC 19, which is composed of benzyl alcohol, 1,3-bis(aminomethyl)benzene, 3-amino-methyl-3,5,5-trimethylcyclohexylamine and BPA-epichlorohydrin polymer. The materials based on the 50% and 75% blends of glycidylated catechin exhibited slightly lower and 10 °C higher *T*_g_ than DGEBA-based materials, respectively. These results may seem surprising as the introduction of tetra-epoxy monomers with the catechin rigid structure was expected to increase glass transition temperature. However, the role of the di-epoxy benzodioxane derivative is not elucidated and may explain these results.

#### 5.2.5. Conclusions

A wide range of di-aromatic bio-based epoxy monomers has been synthesized and cured over the past years with most of them exhibiting interesting and varied properties in terms of glass transition temperature and thermal stability. Shrewd strategies have been developed to slightly or largely step away from the detrimental structure of BPA, but similarly to mono-aromatic compounds, very few were studied for their estrogen activity. The following tables (Table 3, Table 4 and Table 5) gather the known data on cured materials based on the di-aromatic di- and poly-epoxy monomers.

### 5.3. Polyaromatic Epoxy Compounds

As observed in the previous parts, it is hard to match up with the properties of DGEBA-based materials, especially in term of glass transition temperature, thus making the use of polyaromatic and possibly poly-epoxy derivatives a viable alternative. Moreover, the direct use of the biomass would avoid the costly and time-consuming depolymerization and purification steps. The next part will present the epoxy prepolymers containing at least three aromatic moieties and two or more epoxy rings.

#### 5.3.1. Glycidylated Lignin

Chemical modifications of lignin functional groups were carried out to increase its solubility and chemical reactivity, and thus its range of applications. Among the possible functionalizations, modification of the hydroxyl groups is the most versatile since phenolic hydroxyl groups are the most reactive groups and can significantly affect the chemical reactivity of the materials. Allylation [178], esterification [179,180], phenolation [181] or etherification [172,182,183,184] of lignin have been widely investigated. However, only few research groups attempted to epoxidize raw depolymerized lignin by direct *O*-glycidylation.

As the isolation of pure building blocks from lignin is both economically and environmentally costly, Fache et al. [185] developed model mixtures of depolymerization products of lignin from both hardwood and softwood, including for example vanillin, syringaldehyde and their acid equivalents. After a Dakin oxidation to increase the number of reactive hydroxyl groups and an *O*-glycidylation step, the materials cured with IPDA showed interesting thermo-mechanical properties, with *T*_g_ values of 99 °C and 113 °C for hardwood and softwood model mixtures, respectively. These temperatures remain lower than that of DGEBA-based materials synthesized by the same authors in fairly similar conditions [135], probably due to the mono-epoxy derivatives included in the mixtures. Both materials exhibited a good thermal stability with onset degradation temperatures above 250 °C under nitrogen atmosphere and high char yields of 20%–23% at 600 °C. This study demonstrated that purification steps are not necessarily required to achieve high-performance epoxy thermosets from biomass. 

Ferdosian et al. [186] carried out the glycidylation of de-polymerized organosolv or Kraft lignin with epichlorohydrin and highlighted the presence of epoxy by normalized FTIR. Then, they prepared materials of the resulting epoxidized lignin by curing with aromatic and aliphatic amines to show the influence of the curing agent on the thermal stability. Despite the good thermal stability and very high char rate at 800 °C, no glass transition temperature or mechanical properties were given.

Following the work of Zhao et al. [187], Sun et al. [188] prepared a liquid epoxy resin from phenolated lignosulfonate and focused their study on its curing kinetics with maleic anhydride. No data on the material properties were given.

**Figure 28:**
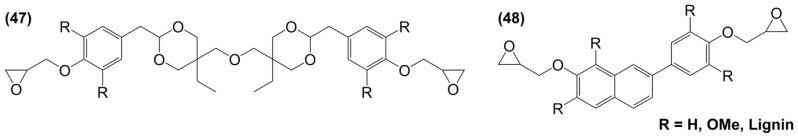
Lignin-based epoxy resins synthesized by Kaiho et al. [189].

Kaiho et al. [189] used a three-steps pathway to synthesize lignin-based epoxy resins (Figure 28): (i) selective β-*O*-4 bond cleavage of *eucalyptus globulus* lignin, (ii) transacetalization for the synthesis of spiro rings (**47**) or annulation to form a phenyl-naphthalene derivative (**48**) and (iii) *O*-glycidylation with epichlorohydrin. The obtained resins were cured with phenol novolac yielding materials with glass transition temperatures ranging from 67 to 134 °C and of 95 °C for an epoxy/bisphenol A system. As expected from the structures, the more rigid structure of the phenyl-naphthalene lignin epoxy prepolymers lead to higher *T*_g_ values and higher flexural strength.

Similarly, Asada et al. [190] used various low molecular weight lignins extracted with methanol to synthesized bio-based epoxy resins. The glycidylation was assessed by FTIR and ^1^H NMR spectroscopies and four sets of materials were cured using two epoxy resins: glycidylated lignin and an oil-based commercial Epikote 828 (EP828, DGEBA from Japan Epoxy Resins Co. Ltd., Nagoya, Japan) and two phenolic hardeners: non-glycidylated lignin and TD2131 (a phenol novolac from DIC Corp., Tokyo, Japan). The materials exhibited high thermal stabilities with *T*_d,5%_ ranging from 259 to 326 °C, still lower than the fully oil-based material (361 °C) but with higher char rates, up to 41% at 800 °C. Unfortunately, no glass transition temperatures or mechanical properties were provided. It is worth noting that the lignin extract insoluble in methanol was enzymatically transformed into glucose, increasing the biomass promotion.

Other uses have been found to lignin for the improvement of epoxy resins. Lignin functionalized by a dicarboxylic acid prepared by dimerizing unsaturated fatty acids was considered as a phenolic aldehyde amine curing agent by Fang et al. [191]. Similarly, Engelmann et Ganster [192] blended a low molecular weight lignin fraction with the bio-based tri-glycidylated glycerol for the preparation of composites reinforced with cellulosic fibers.

#### 5.3.2. Glycidylated Tannins

Few research papers are devoted to the use of tannins and tannin-based molecules for epoxy materials synthesis compared to lignin ones. Maybe the reduced availability of the former is a key factor in this result, as the complexity and the variability of both these bio-resources seem similar. As previously mentioned before, Aouf et al. [176] considered the hydrolysable tannins from tara as potential precursor for epoxy prepolymer synthesis, but they first carried out a depolymerization to obtain gallic acid mixtures prior to glycidylation. Benyahya et al. [193] focused their work on the inexpensive green tea (*Camellia sinensis*) tannins, which are condensed tannins mainly composed of epicatechin, catechin dimers and their galloylated equivalents. The tannins extracts were glycidylated with epichlorohydrin and cured with isophorone diamine. The obtained materials exhibited a *T*_α_ of 142 °C, a value similar to the 140 °C of the material from IPDA and DER352 (a mixture of DGEBA and DGEBF supplied by Dow (France)). Swelling ratios and soluble fractions of these materials also proved to be very low. Jahanshahi et al. [194,195] recently published two papers on the use of condensed tannins for the synthesis of bio-based materials. In the first one [194], Mimosa (*Acacia mearnsii*) bark tannins were glycidylated with epichlorohydrin but the epoxy rings were then reacted with acrylic acid for adhesive synthesis. On the contrary, in their second paper, they focused their efforts on the glycidylation optimal parameters and characterization of the epoxy prepolymers, thus very brief results on tensile shear tests of glycidylated tannins mixed with DGEBA were mentioned.

#### 5.3.3. Glycidylated Cardanol

**Figure 29:**
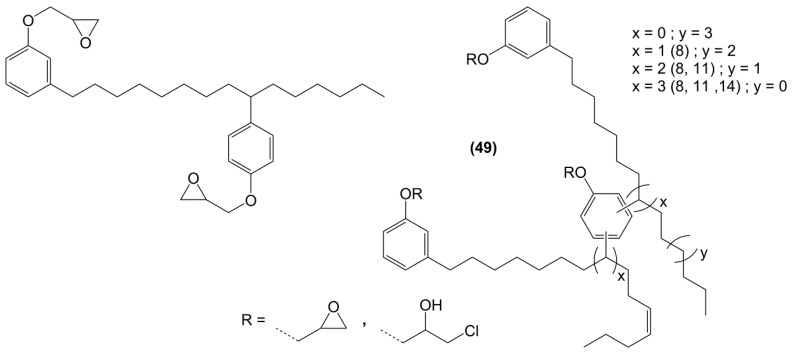
Idealized structure of phenolated cardanol (left) and real structure (**49**) of NC514 by Cardolite. The numbers between brackets indicate the locations of the phenol moieties along the aliphatic alkyl chain.

To increase the low glass transition temperatures obtained with materials based on glycidylated cardanol, a polyaromatic derivative of it can be designed by a two-steps synthesis, beginning by a phenolation step and followed by direct *O*-glycidylation. The resulting product is idealized by a diepoxidized diaromatic cardanol structure and marketed with the name NC514 by Cardolite. However, Jaillet et al. [196] showed that the commercial product is a mixture (**49**), in which chains are crosslinked and some epoxy rings are opened, as shown in Figure 29. This product may cause an allergic skin reaction (H317), but can be used as food contact material, according to the supplier.

Epoxy-amine based materials showed moderate glass transition temperature, from 13 to 30 °C with linear aliphatic diamines [197,198,199]. The highest *T*_g_ was reached when epoxidized cardanol was cured with isophorone diamine, up to 50 °C, which is still 100 °C lower than that of DGEBA-based material [196]. Nevertheless, the fatty chain of the epoxidized cardanol allows more flexibility, conferring mechanical properties suitable for particular coating applications. Glycidyl ether groups have also been converted to methacrylate, allowing radical polymerization for other coating applications [200,201].

#### 5.3.4. Other Bio-Based Poly-Aromatic Monomers

**Scheme 14:**
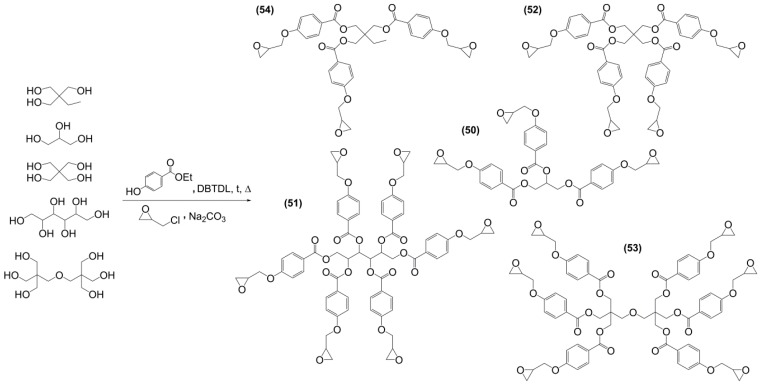
Synthetic pathway to obtain poly-aromatic poly-glycidyl ethers developed by Fourcade et al. [167].

Fourcade et al. [167] applied the same strategy as previously described (see Section 5.2.2.) on various aliphatic polyols (Scheme 14). Glycerol (**50**) and sorbitol (**51**) are bio-based precursors, pentaerythritol (**52**), di-pentaerythritol (**53**) and trimethylolpropane (**54**) are obtained via formaldehyde and acetaldehyde from syngas of the Biomass-to-Liquid (BtL) process or carbon monoxide hydrogenation. Moreover, according to their corresponding MSDS files, these polyols and ethyl-4-hydroxybenzoate show no toxic risk. Only the dibutyltin dilaurate (DBTDL), which is used as catalyst in the phenolation step and the unavoidable epichlorohydrin present safety risks.

The phenolation step allowed 75% to 100% of conversion and the glycidylation of the resulting phenolic compounds led to an overall functionality ranging between 2 and 5. The synthesized polyaromatic glycidyl epoxides were blended with DGEBA and cured with dicyandiamide. From a general point of view, the addition of these new resins decreased the glass transition temperature of the blend materials, up to 24% when 25% are used. Glycidyl ether of trimethylolpropane-tris(4-hydroxybenzoate) was cured with dicyandiamide in the absence of DGEBA and the corresponding material showed a *T*_g_ of 105 °C, slightly lower than that of DGEBA-based material (119 °C). This result is very promising since materials can be prepared from non-toxic hydroxyl compounds and almost meet the thermal properties of the harmful DGEBA-based materials.

**Figure 30:**
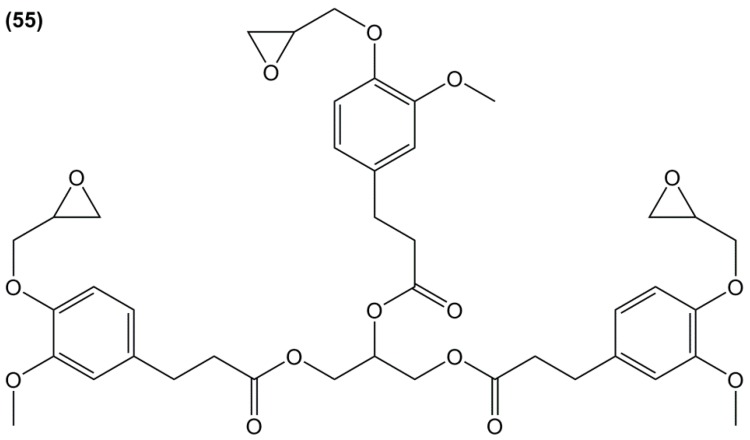
Tri-epoxy monomer based on ferulic acid and glycerol synthesized by Ménard et al. [166].

Similarly, Ménard et al. [166] synthesized a tri-aromatic tri-epoxy monomer based on glycerol and ferulic acid (**55**) (Figure 30), although the esterification step was carried out with an enzymatic catalyst. After curing with IPDA, difurfurylamine and 1,10-diaminodecane, the thermosets showed *T*_g_ and *T*_α_ values ranging from 54 to 73 °C and 67 to 92 °C, respectively, with lowest values observed for the aliphatic diamine. These values remain significantly lower than those observed for DGEBA-based materials, as the increased functionality does not compensate the effect of the long aliphatic segments.

**Figure 31:**
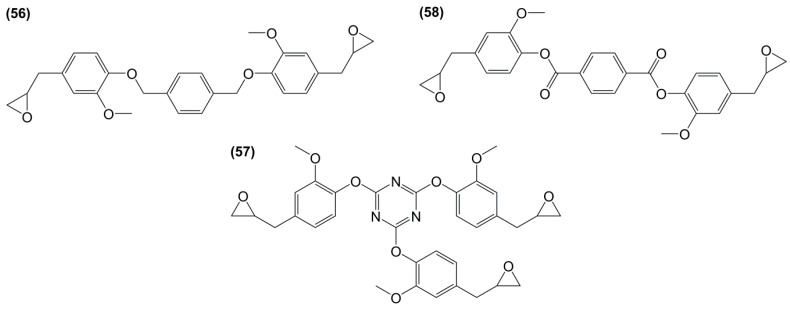
Eugenol-based poly-aromatic epoxy monomers.

Very recently, Wan et al. [121,202] prepared two eugenol-based di-epoxy compounds, using a two-steps synthesis. The first step consisted in a Williamson etherification reaction between the hydroxyl group of eugenol and chloride aromatic derivatives, α,α′-dichloro-*p*-xylene [202] or cyanuric chloride [121]. The resulting allylated intermediate was achieved with an almost quantitative yield. Then, the carbon-carbon double bonds were oxidized using *m*CPBA with a moderate yield (44%–70%), yielding di- (**56**) and tri-glycidylated (**57**) derivatives (Figure 31). Despite a high net bio-based content (70%) of these new epoxy resins, the chosen synthetic pathway requires the use of *m*CPBA, which is known to be dangerous, and chlorinated derivatives, which are highly toxic (H302-H314-H330) for cyaniric chloride and detrimental for the environment (H400) for α,α′-dichloro-*p*-xylene.

Both eugenol-based epoxies showed lower reactivity towards amine than DGEBA, due to the lower electron withdrawing effect of the -CH_2_Ph epoxy-ring, compared to the usual -CH_2_OPh in BPA, but they avoid the use of epichlorohydrin and still, eugenol-based materials were successfully prepared by curing with aromatic diamines. The material based on the diglycidyl ether compounds prepared from α,α′-dichloro-*p*-xylene showed a *T*_g_ 40 °C lower than for DGEBA-based materials, probably due to the methoxy groups on the aromatic rings and the -CH_2_O linkages between them. For the material obtained from cyanuric chloride, the glass transition temperature proved to be 33 °C higher despite the methoxy moieties, probably thanks to the tri-functional nature of the prepolymer.

Wan et al. [120] also prepared a bio-based epoxy building block in a two-step synthesis from eugenol and terephthaloyl chloride: (i) a coupling reaction between eugenol and terephthaloyl chloride, followed by (ii) an epoxidation by oxidation of the C=C double bonds (**58**). The coupling step allowed 93% of yield and the final product was a white solid. However, the high toxicity of the commercially available terephthaloyl chloride has to be taken in account. The material obtained by curing with 3,3′-diaminodiphenyl sulfone showed lower *T*_g_ and thermal stability and slightly higher mechanical properties than the one based on DGEBA. Furthermore, it exhibited interesting intrinsic flame retardancy.

**Figure 32:**
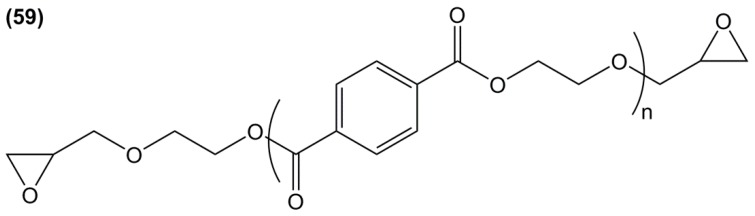
Epoxy monomers obtained from glycolyzed PET waste by Czub [203].

Czub [203] considered recycling polyethylene terephthalate (PET) wastes, especially coming from bottles, for the synthesis of epoxy resins (**59**). The precursor of PET, terephtalic acid, is mostly produced by air oxidation of oil-based *p*-xylene [204]. However, as previously stated, *p*-xylene can be obtained from cellulosic biomass, for example via dehydration of *iso*-butanol into *iso*-butene, dimerization into *iso*-octene and catalytic conversion. It can also be synthesized by dehydrogenation of 3-carene extracted from pine tree or limonene extracted from citrus fruits into *p*-xylene. Thus, the author degraded PET waste by glycolysis reaction into small aromatic hydroxy telechelic oligomers. Using epichlorohydrin, these polyhydroxyl derivatives were glycidylated and mixed with DGEBA oligomers leading to an improved water absorption and resistance to HNO_3_, H_2_SO_4_ and ethyl acetate (Figure 32). The addition of 5%–20% of glycidylated PET-wastes in oligo(DGEBA) cured with isophorone diamine led to similar and slightly higher *T*_g_ values, from 56 °C to 53–63 °C measured in DMTA.

#### 5.3.5. Conclusions

Due to their complexity and variability, bio-mass resources such as lignin or tannins have not received as much attention as smaller molecules for BPA substitution. However, as observed in the previous parts, they have been successfully turned into poly-epoxy monomers and used for the synthesis of materials exhibiting interesting properties. Similarly, smaller bio-based molecules such as eugenol and ferulic acid were turned into poly-aromatic epoxy monomers, but still few materials reached the high *T*_g_ values of their DGEBA counterparts. These results are gathered in Table 6 and Table 7.

## 6. General Conclusions

Biorefinery processes and retreatment of biomass was out of our scope. However, it is clear that the future of bio-based polymers, including building blocks for thermosetting epoxies is strongly dependent on the future of biorefinery. The goal of a biorefinery is to produce high quality chemicals for fuels, monomers, and finally polymers and materials. New screening techniques like “Green Chemistry” and techno-economic and Life Cycle Assessment (LCA) indicators can help chemical-based biorefinery project developments. But efficient, economical, and large-scale synthesis of monomers is crucial, and the first key parameter remains a resource pool that should be abundant and easily accessed.

At first glance, there are a lot of opportunities to develop molecular biomass and monomers for preparation of renewable epoxy formulations and materials. But, the biggest changes for thermosetting epoxy formulations in the near future will certainly come from the regulation changes like the environmental directives for reducing Volatile Organic Constituents (VOC), the “Restriction of certain Hazardous Substances” (RoHS), or the “Registration, Evaluation and Authorisation of Chemicals”, (REACh). Bio-based compounds will not be the only solution for these policy pressures but will be an opportunity.

There are many different routes to synthesize bio-based aromatic epoxy monomers and hence to replace DGEBA. However, we have to take into account several parameters such as the epoxy functionality; the different applications—for example coatings are totally different from composites and the adaptation of properties to applications. Hence, even if DGEBA is very interesting since it allows to cover all applications, it seems difficult to imagine to replace DGEBA by the same bio-based epoxy monomer in all applications. Currently, only a handful of patents led to commercialization of products, such as epoxided cardanol or vegetable oils. However, these epoxide monomers led to low *T*_g_ polyepoxide networks for low *T*_g_ applications. Epoxy aromatic monomers leading to high *T*_g_ polyepoxide are desired and correspond to an important challenge. Some industrial countries have declared bisphenol A (BPA) to be a toxic substance that causes risks to human health as well as to the environment; thus it has to be banned for all food contact applications in the next coming years, and in the immediate future the pressure is on its replacement. BPA is industrially produced from condensation of acetone with phenols. Therefore, bio-based aromatic/rigid epoxy monomers are still needed in order to fulfil good compromise between processing and properties, and able to enable replacement of BPA.

Developing highly efficient, safe, low waste, low toxicity and atom economy processes are the key words for green chemistry. Chemistry has to be simple, practical, and operational, and catalysts are expected to play an important role. Epichlorohydrin is the preferred way to prepare epoxy monomers; but epichlorohydrin is a toxic molecule which has to be manipulated in a safe environment. Epoxidation without the use of epichlorohydrin is a key challenge. Even if allylation or crotonization of alcohols can be a first step for epoxydation, the second step, i.e., double bond oxidation, requires the use of hazardous catalysts. Therefore, the oxidation of terminal double bonds is an interesting route but still needs research to propose industrial routes.

## Figures and Tables

**Table 1:** Mono-aromatic, di-epoxy monomers and thermal properties of the cured materials. *T*_g_ values are indicated in plain text, *T*_α_ are in italics.

Epoxy	Curing Agent	*T*_g_ (°C) or *T*_α_ (°C)		*T*_d_,_5%_ (°C)	Reference
		Materials	DGEBA Comparison		
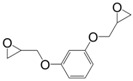	2,5-difluoroterephthalic acid	69	66	242	[154]
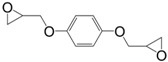	2,5-difluoroterephthalic acid	74	66	305	
	Diethyl toluene diamine EPIKURE W	*193* ^a^	*185* ^a^*/198* ^b^	-	[150]
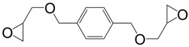	4,4′-methylene biscyclohexylamine PACM	*100* ^a^*/111* ^b^	*167* ^a^*/176* ^b^	-	
	Diethyl toluene diamine EPIKURE W	*132* ^a^*/140* ^b^	*185* ^a^*/198* ^b^	-	
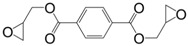	Methyhexahydrophthalic anhydride MHHPA	*129* ^b^	*125*	284	[132]
	Poly(propylene glycol) bis(2-aminopropyl ether) D230	*92* ^b^	97	266	
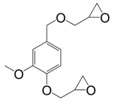	4,4′-methylenebiscyclohexylamine	*100* ^a^*/107* ^b^	*149* ^a^*/158* ^b^	341	[17]
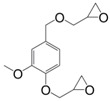	Isophorone Diamine	*97/106 ^b^*	*166/182 ^b^*	361 ^c^	[27,135]
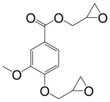	Isophorone Diamine	152/*166* ^b^	166/*182* ^b^	315 ^c^	
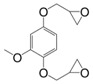	Isophorone Diamine	132/*154* ^b^	166/*182* ^b^	328	
	Bisfurfurylamine A	85	111	312	[148]
	Vanillylamine	64	67	262	
	1,10-diaminodecane	74	98	328	
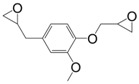	Hexahydrophthalic anhydride	114	106	321	[119]
	Maleopimaric acid	155	-	317	
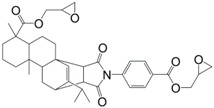	1,2-cyclohexanedicarboxylic anhydride	154	144	311	[139]

^a^ *T*_α_ measured by DMA at the peak position of loss modulus curve; ^b^ *T*_α_ measured by DMA at the maximum of tan δ; ^c^ T_d,50%_.

**Table 2:** Mono-aromatic, poly-epoxy resins and thermal properties of the cured materials. *T*_g_ values are indicated in plain text, *T*_α_ are in italics.

Epoxy	Curing Agent	*T*_g_ (°C) or *T_α_* (°C)		*T*_d,5%_ (°C)	Reference
		Materials	DGEBA Comparison		
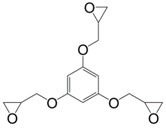	PE-C9-NH2	*102* ^a^	57	266 ^c^	[141]
	PE-C18-NH2	*112* ^a^	52	283 ^c^	
	Isophorone Diamine	177	157	306 (10%)	[142]
	Decane-1,10-diamine	137	-	300 (10%)	
	Difurfuryl amine	134	-	290 (10%)	
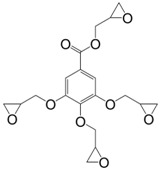	Isophorone Diamine	*233 ^a^*	160	300	[22]
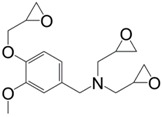	Isophorone Diamine	176/*194* ^b^	166/*182* ^b^	308	[148]
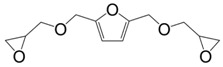	4,4′-metylene biscyclohexylamine PACM	*71* ^a^*/80* ^b^	*167* ^a^*/176* ^b^	303	[150]
	Diethyl toluene diamine EPIKURE W	*88* ^a^*/94* ^b^	*185* ^a^*/198* ^b^	-	
	5,5′-methylene difurfurylamine	*56* ^a^*/62* ^b^	*121* ^a^*/128* ^b^	272	[151]
	5,5′-ethylidene difurfurylamine	*56* ^a^*/69* ^b^	*128* ^a^*/142* ^b^	272	
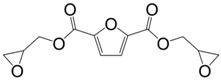	methyhexahydrophthalic anhydride MHHPA	*152* ^b^	125	293	[132]
	poly(propylene glycol) bis(2-aminopropyl ether) D230	101 ^b^	97	267	
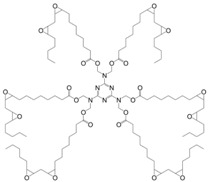	4-methyl hexahydrophthalic anhydride	78–92	-	246–316	[153]

^a^ *T_α_* measured by DMA at the peak position of loss modulus curve; ^b^ *T*_α_ measured by DMA at the maximum of tan δ; ^c^ *T*_d_ under air flow.

**Table 3:** Di-epoxy resins with two aromatic rings separated by one atom, and thermal properties of the cured materials.

Epoxy	Curing Agent	*T*_g_ (°C) or *T*_α_ (°C)		*T*_d,5%_ (°C)	Reference
		Materials	DGEBA Comparison		
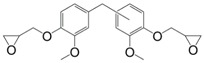	4,4′-methylene-biscyclohexylamine	*104* ^a^*/111* ^b^	*149* ^a^*/158* ^b^	363	[17]
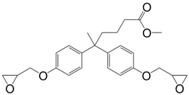	Isophorone Diamine	158	165	361	[157]
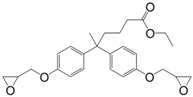	Isophorone Diamine	136		363	
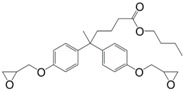	Isophorone Diamine	96		363	
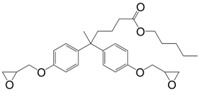	Isophorone Diamine	86		362	
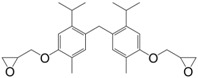	4,4′-diaminodiphenyl methane	161	179	367/368 ^c^	[116]
	4,4′-methylene bis(5-isopropyl-2-methylaniline)	143	154	363/360 ^c^	
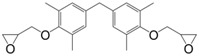	4,4′-diaminodiphenyl methane	184/*183* ^b^	-	346/355 ^c^	[159]

^a^ *T_α_* measured by DMA at the peak position of loss modulus curve; ^b^ *T_α_* measured by DMA at the maximum of tan δ; ^c^ *T*_d_ under air flow.

**Table 4:** Di-epoxy monomers with two aromatic rings separated by more than one atom exhibiting ester or amide bonds, and thermal properties of the cured materials.

Epoxy	Curing Agent	*T*_g_ (°C) or *T_α_* (°C)		*T*_d,5%_ (°C)	Reference
		Materials	DGEBA Comparison		
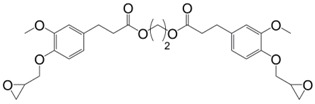	Isophorone diamine	*66* ^b^	*170* ^b^	300	[123]
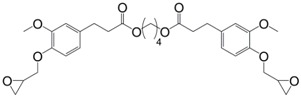	Isophorone diamine	51/*61* ^b^	150/*174* ^b^	314	[166]
	1,10-diaminodecane	33/*33* ^b^	98/*97* ^b^	314	
	Difurfurylamine	32/*41* ^b^	92/*110* ^b^	306	
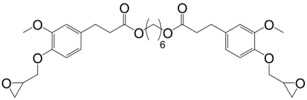	Isophorone diamine	*50* ^b^	*170* ^b^	331	[123]
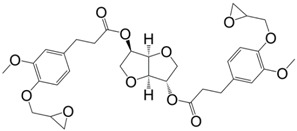	Isophorone diamine	85/*99* ^b^	150/*174* ^b^	295	[166]
	1,10-diaminodecane	54/*68* ^b^	98/*97* ^b^	292	
	Difurfurylamine	63/85 ^b^	92/110 ^b^	304	
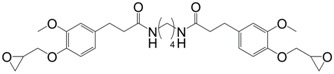	Isophorone diamine	75	150	282	
	1,10-diaminodecane	69	98	297	
	Difurfurylamine	74	92	271	

^a^ *T_α_* measured by DMA at the peak position of loss modulus curve; ^b^ *T_α_* measured by DMA at the maximum of tan δ; ^c^ *T*_d_ under air flow.

**Table 5:** Di-epoxy monomers with two aromatic rings separated by more than one atom, and thermal properties of the cured materials.

Epoxy	Curing Agent	*T*_g_ (°C) or *T_α_* (°C)		T_d,5%_ (°C)	Reference
		Materials	DGEBA Comparison		
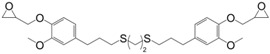	4,4′-Diamino diphenyl methane	60/70	-	-	[165]
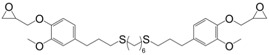	4,4′-Diamino diphenyl methane	45/58	-	-	
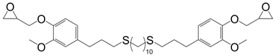	4,4′-Diamino diphenyl methane	39/54	-	-	
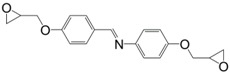	4,4′-Diamino diphenyl methane	200	-	341/342 ^c^	[16]
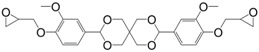	Hexahydrophthalic anhydride	*134* ^b^	*132* ^b^	-	[173]
	Phthalic anhydride	*138* ^b^	*-*	-	
	Nadic anhydride	*147* ^b^	*-*	-	
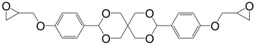	Hexahydrophthalic anhydride	*170* ^b^	*132* ^b^	-	
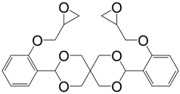	Hexahydrophthalic anhydride	*149* ^b^	*132* ^b^	-	

^a^ *T_α_* measured by DMA at the peak position of loss modulus curve; ^b^ *T_α_* measured by DMA at the maximum of tan δ; ^c^ *T*_d_ under air flow.

**Table 6:** Poly-aromatic epoxy monomers from crude bio-mass and thermal properties of the cured materials.

Epoxy	Curing Agent	*T*_g_ (°C) or *T*_α_ (°C)		*T*_d,5%_ (°C)	Reference
		Materials	DGEBA Comparison		
Epoxidized depolymerized kraft lignin	Diethylenetriamine	-	-	252 ^d^	[186]
	4,4-diaminodiphenyl methane	-	-	290 ^d^	
Epoxidized depolymerized organosolv lignin	Diethylenetriamine	-	-	228 ^d^	
	4,4-diaminodiphenyl methane	-	-	257 ^d^	
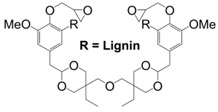	Phenol Novolac	94	95	-	[189]
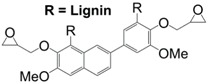		134		-	
Epoxidized lignin (Cedar)	Phenol Novolac (TD2131)	-	-	293	[190]
	Lignin (Cedar)	-	-	296	
Epoxidized lignin (Eucalyptus)	Phenol Novolac (TD2131)	-	-	275	
	Lignin (Eucalyptus)	-	-	274	
Epoxidized lignin (Bamboo)	Phenol Novolac (TD2131)	-	-	266	
	Lignin (Bamboo)	-	-	259	
Epoxidized green tea extract	Isophorone Diamine	*142* ^a,b^	*140* ^a,b^	256/267 ^c^	[193]
Epoxidized cardanol (NC514)	Isophorone Diamine	*50* ^a,b^	*155* ^a,b^	350	[205]
	Jeffamine T403	23	70	352 ^e^	[198]
	Isophorone Diamine	41	121	350 ^e^	
		50/*59* ^b^	158/*158* ^b^	366 ^e^/363 ^c,e^	[196]
	Jeffamine D400	15/*9* ^b^	-	362 ^e^/361 ^c,e^	
	PE-C9-NH2	13	279	-	[141]
	PE-C18-NH2	14	284	-	
	Phenalkamine NX5454	30/*38* ^a,b^	-	325 ^f^/322 ^c,f^	[206]
	Cardanol cysteamine	19/*21* ^a,b^	-	328 ^f^/311 ^c,f^	

^a^ *T_α_* measured by DMA at the peak position of loss modulus curve; ^b^ *T_α_* measured by DMA at the maximum of tan δ; ^c^ *T*_d_ under air flow; ^d^ IDT = initial decomposition temperature; ^e^ *T*_d,30%_; ^f^ *T*_d,10%_.

**Table 7:** Poly-aromatic di- and tri-epoxy monomers and thermal properties of the cured materials.

Epoxy	Curing Agent	*T*_g_ (°C) or *T*_α_ (°C)		*T*_d,5%_ (°C)	Reference
		Materials	DGEBA Comparison		
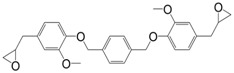	4,4′-diaminodiphenyl methane	*114* ^b^	*154* ^b^	341	[202]
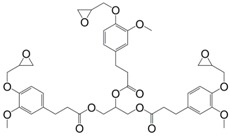	1,10-diaminodecane	54/*70* ^b^	150/*174* ^b^	293	[166]
	Isophorone Diamine	73/*92* ^b^		294	
	Difurfurylamine	58/*67* ^b^		284	
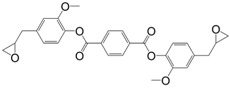	3,3′-diaminodiphenyl sulfone	*168* ^b^	*174* ^b^	338/337 ^c^	[120]
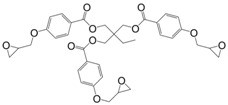	Dicyandiamide	*105* ^b^	*119* ^b^	305 ^c^	[167]
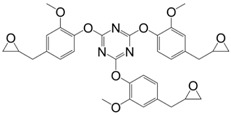	3,3′-diaminodiphenyl sulfone	*207* ^b^	*174* ^b^	326	[121]

^a^ *T_α_* measured by DMA at the peak position of loss modulus curve; ^b^ *T_α_* measured by DMA at the maximum of tan δ; ^c^ *T*_d_ under air flow.
